# Deep-learning side-channel analysis of embedded ML-KEM: Single-trace message recovery, cross-device generalization, and frequency-band leakage characterization

**DOI:** 10.1371/journal.pone.0358974

**Published:** 2026-11-04

**Authors:** Chung-Wei Kuo, Yu-I Hung, Kai-Lun Lin

**Affiliations:** 1 Department of Information Engineering and Computer Science, Feng Chia University, Taichung, Taiwan; 2 Master’s Program of Information and Communication Security, Feng Chia University, Taichung, Taiwan; China Medical University Taiwan, TAIWAN

## Abstract

Quantum computing undermines traditional public-key cryptosystems like RSA and ECC, prompting NIST to standardize Module Lattice Key Encapsulation Mechanism (ML-KEM) as a core Post-Quantum Cryptography (PQC) scheme in August 2024. Despite its theoretical security, ML-KEM’s practical vulnerability in embedded systems remains underexplored. This study evaluates ML-KEM on an ARM Cortex-M4 microcontroller under deep learning(DL)-based side-channel attacks (DL-SCA), revealing that a single power trace enables 32-byte message recovery with 99.98% accuracy, while cross-device testing across three chips achieves over 99% accuracy, highlighting critical DL-driven threats. To better understand the spectral characteristics exploited by deep-learning-based side-channel attacks, we conduct a controlled frequency-band sensitivity study using Butterworth and Chebyshev low-pass filters applied to recorded traces. Across different cutoff frequencies and filter orders, the validation accuracy decreases substantially under aggressive attenuation, indicating that a significant fraction of exploitable leakage resides in higher-frequency components. Rather than claiming that offline digital filtering constrains a realistic adversary, we interpret these results as evidence for where leakage-sensitive information is concentrated, thereby providing guidance for implementable countermeasures such as masking, shuffling, desynchronization, and acquisition-path power/EM conditioning in embedded PQC systems. This cost-efficient solution requires no algorithm/hardware modifications, emphasizing the urgency of implementation-level protections for secure PQC deployment in resource-constrained systems.

## 1. Introduction

Digital technologies ranging from electronic banking and cloud file storage to instant messaging have become indispensable to modern consumer and industrial ecosystems. These services rely on public-key cryptography (PKC) to protect sensitive data and communications [1,2]. For decades, widely deployed PKC schemes such as RSA (based on the hardness of large-integer factorization) and elliptic curve cryptography (ECC, based on the discrete logarithm problem) have secured critical infrastructure and global transactions under classical computing assumptions. This foundation is now threatened by advances in quantum computing. Shor’s algorithm, introduced in 1994 [3], showed that sufficiently powerful quantum computers can solve integer factorization and discrete logarithms in polynomial time, undermining the security of RSA and ECC. To address this risk, PQC has emerged as a major research focus. PQC schemes are built on mathematical problems, such as lattice-based hardness assumptions, that are believed to resist both classical and quantum attacks. In August 2024, the U.S. National Institute of Standards and Technology (NIST) standardized the Module-Lattice Key Encapsulation Mechanism (ML-KEM) [4], derived from the Module Learning with Errors (M-LWE) problem, due to its strong security properties and practical performance. While ML-KEM offers robust algorithmic security, its implementations on resource-constrained embedded platforms, such as IoT microcontrollers, smart cards, and mobile devices, remain susceptible to side-channel analysis (SCA) [5]. Such devices can unintentionally leak measurable physical signals (e.g., power consumption [6], electromagnetic (EM) radiation [7], timing variations, and temperature fluctuations [8]) that may reveal sensitive information about the cryptographic computation. Since Kocher’s seminal work in 1996 [5], extensive research has shown that implementation-level leakage can expose secret keys, intermediate states, or plaintext, making practical security as critical as mathematical design. The rapid adoption of deep learning has further intensified the SCA threat landscape. Unlike traditional SCA pipelines that depend heavily on handcrafted features and expert-driven signal processing, DL-based profilers can automatically extract discriminative patterns from noisy traces, often tolerating measurement noise and mild misalignment. This capability lowers the technical barrier for attackers and can substantially improve attack effectiveness. Recent studies have reported near-perfect recovery of secret information in certain settings, including scenarios where only a single power trace is available. For embedded PQC deployments commonly found in IoT and edge computing, these developments create an urgent need to understand practical leakage sources and to identify mitigation strategies that remain feasible under tight resource constraints.

In this work, we study implementation-level leakage in embedded ML-KEM from three complementary perspectives. First, we demonstrate that a profiling attack can recover the 32-byte encapsulated message from a single power trace on a Cortex-M4 platform, showing that the poly_frommsg() stage remains highly vulnerable despite constant-time coding practices.Second, we evaluate whether the learned leakage features transfer across nominally identical devices, thereby assessing the practical realism of profiling attacks beyond a single laboratory chip.Third, we use controlled low-pass filtering not as a standalone defense claim, but as an analysis tool to identify which spectral regions most strongly support attack success.Taken together, these three elements position the present work not merely as another deep-learning side-channel case study, but as an investigation of single-trace recoverability, cross-device transferability, and leakage frequency dependence in embedded ML-KEM implementations.The main novelty of this work is that it combines single-trace message recovery with cross-device analysis, giving a more complete and practical view of side-channel threats in embedded PQC implementations.

This analysis provides evidence that a substantial portion of exploitable features resides in higher-frequency bands and, importantly, helps inform the design and selection of implementable mitigations (e.g., masking/shuffling, desynchronization, and board-level or on-chip power/EM filtering in the physical acquisition path).

The main contributions of this work are summarized as follows:

(a) We demonstrate single-trace recovery of the 32-byte encapsulated message from an embedded ML-KEM implementation on Cortex-M4, achieving near-perfect byte-level accuracy under a profiling-based DL-SCA setting.
(b) We quantify cross-device transferability across three additional STM32F415 devices and show that, although byte-level success remains consistently high, full-message recovery is substantially more sensitive to device-level variation.
(c) We perform a frequency-band sensitivity analysis using Butterworth and Chebyshev low-pass filtering to characterize which spectral components most strongly contribute to learnable leakage.
(d) Based on these observations, we discuss how the measured leakage dependence can inform realistic implementation-level protections, including masking, shuffling, desynchronization, and physical acquisition-path filtering.

The remainder of this paper is organized as follows. Chapter 2 reviews related work on ML-KEM, SCA, and DL-based attacks, as well as the background on PQC and low-pass filtering. Chapter 3 presents the research methodology, including trace collection, model architecture, and defense implementation. Chapter 4 reports experimental results, focusing on attack performance and the effectiveness of the filtering countermeasures. Chapter 5 concludes with key findings and future research directions.

## 2. Background

### 2.1 ML-KEM

The Module-Lattice-based Key Encapsulation Mechanism (ML-KEM), also known as CRYSTALS-Kyber, has now been made the standard for Post-Quantum Cryptography (PQC) public-key encryption (PKE) and key-encapsulation mechanism (KEM). ML-KEM is based on the Module Learning-with-Errors (M-LWE) problem and is better than other systems, such as NewHope, which is based on the Ring Learning-with-Errors (R-LWE) problem [9]. At equivalent security levels, ML-KEM achieves more compact public and private keys, while its polynomial vector dimension k can be tuned to balance efficiency and security.

To accelerate polynomial multiplication, ML-KEM leverages the Number Theoretic Transform (NTT), which maps polynomial coefficients into the NTT domain. This transformation reduces the computational complexity of polynomial multiplication from $𝒪(n^{2})$ to 𝒪(nlogn), and has been one of the key factors behind its adoption as a standardized PQC PKE/KEM scheme.

The ML-KEM.CPAPKE scheme provides IND-CPA security, ensuring robustness against chosen-plaintext attacks. By applying a modified Fujisaki-Okamoto (FO) transform, ML-KEM is upgraded to IND-CCA2 security, thereby providing protection against adaptive chosen-ciphertext attacks.In particular, during the decapsulation process, the recovered random message is re-encrypted and compared with the original ciphertext. This verification step prevents adversaries from exploiting decryption queries, thereby ensuring IND-CCA2 security.

ML-KEM.CPAPKE consists of three core algorithms: Key Generation, Encryption, and Decryption.

In Key Generation, a polynomial matrix 𝐀 and a polynomial vector 𝐬 are sampled. The public key 𝐭 is obtained as the product of 𝐀 and 𝐬, plus an error vector 𝐞. Both 𝐬 and 𝐞 are vectors of *k* polynomials, each containing *n* coefficients, and are transformed into the NTT domain to accelerate multiplication.

In Encryption, a random message *m* is encrypted into a ciphertext *c*, which is composed of two compressed parts: a polynomial vector 𝐮 and a polynomial *v*. Specifically, a 32-byte random message is first expanded into a 256-dimensional polynomial representation before further processing, as illustrated in Algorithm 1 [10].

In Decryption, the ciphertext *c* is decompressed into its original components 𝐮 and *v*. The vector 𝐮 is transformed into the NTT domain and multiplied by the private key 𝐬. Subtracting this result from *v* allows the recovery of the original message *m*. During decapsulation (ML-KEM.CCAKEM.Dec), the decrypted message is re-encrypted and verified against the input ciphertext, again invoking Algorithm 1. This mechanism forms the basis of our side-channel analysis in this study.

ML-KEM is defined in three parameter sets: ML-KEM-512, ML-KEM-768, and ML-KEM-1024, which correspond to security levels approximately equivalent to AES-128, AES-192, and AES-256, respectively. All three variants operate over the same polynomial ring $R_{q}=({\mathbb{Z}}_{q}[X])/(X^{256}+1)$. The detailed parameter configurations are provided in Tables 1 and 2.

**Table 1:** ML-KEM parameter set.

Algorithm	*n*	*q*	*k*	$\eta_{1}$	$\eta_{2}$	$\left(d_{u},d_{v}\right)$	Decryption Failure Rate
ML-KEM-512	256	3329	2	3	2	(10,4)	2^–139^
ML-KEM-768	256	3329	3	3	2	(10,4)	2^–164^
ML-KEM-1024	256	3329	4	2	2	(11,5)	2^–174^

**Table 2:** ML-KEM key and ciphertext sizes (bytes).

Algorithm	Encapsulation Key	Decapsulation Key	Ciphertext	Shared Secret Key
ML-KEM-512	800	1632	768	32
ML-KEM-768	1184	2400	1088	32
ML-KEM-1024	1568	3168	1568	32

**Algorithm 1. Decoding: poly_frommsg()**

**Input:** Input Message in msg[32]

1:  **for** i=0...31 **do**

2:   **for** j=0...7 **do**

3:    b=−((msg[i]≫j) & 1);

4:    r=coeffs[8·i+j];

5:    coeffs[8·i+j]=r⊕(b & (r⊕((q+1)/2)));

6:   **end for**

7:  **end for**

8:  **return** coeffs[]

### 2.2 Side-channel attack

Side-channel analysis exploits physical leakage generated during cryptographic execution to infer sensitive information, such as secret keys or plaintexts. Unlike classical cryptanalysis, which targets the mathematical structure of an algorithm, SCA focuses on implementation-dependent leakage. Common leakage sources include power consumption, electromagnetic radiation, timing variations, temperature fluctuations, and acoustic emissions, as illustrated in Fig 1. Since these physical signals vary with both executed operations and processed data, attackers can analyze them to recover internal secrets, even when the underlying cryptographic algorithm is mathematically secure.

**Fig 1:**
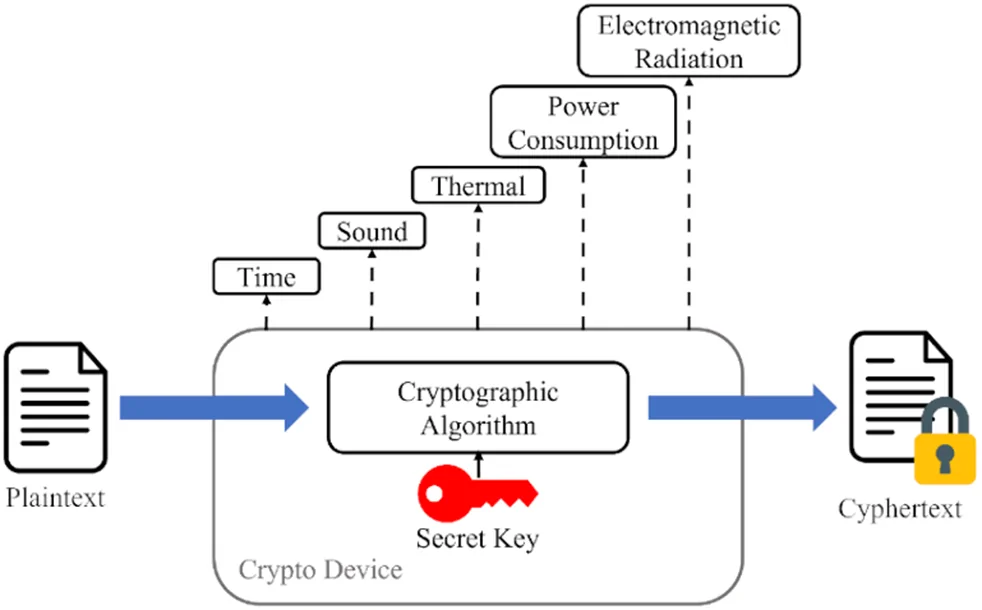
Schematic of device side-channel leakage.

#### 2.2.1 Non-profiling attack.

Non-profiling attacks do not require prior training on a reference device. They mainly exploit either instruction-level leakage, as in Simple Power Analysis (SPA), or data-dependent leakage, as in Differential Power Analysis (DPA) and Correlation Power Analysis (CPA). DPA compares statistical differences between trace groups, while CPA measures correlation between traces and a leakage model such as Hamming weight or Hamming distance. These methods are effective, but usually require a relatively large number of well-aligned traces.

#### 2.2.2 Profiling attack.

Profiling attacks are among the most powerful SCA methods. The attacker first collects labeled traces from a reference device under full control, trains a statistical or machine-learning model, and then applies the trained model to traces from the target device. Traditional template attacks model the leakage distribution of each class, but their performance can degrade under noise or device variation. Deep learning profilers, such as multilayer perceptrons (MLPs) [11] and convolutional neural networks, can learn discriminative features directly from traces and therefore often achieve stronger robustness and attack performance. Fig 2 illustrates the profiling workflow adopted in this study.

**Fig 2:**
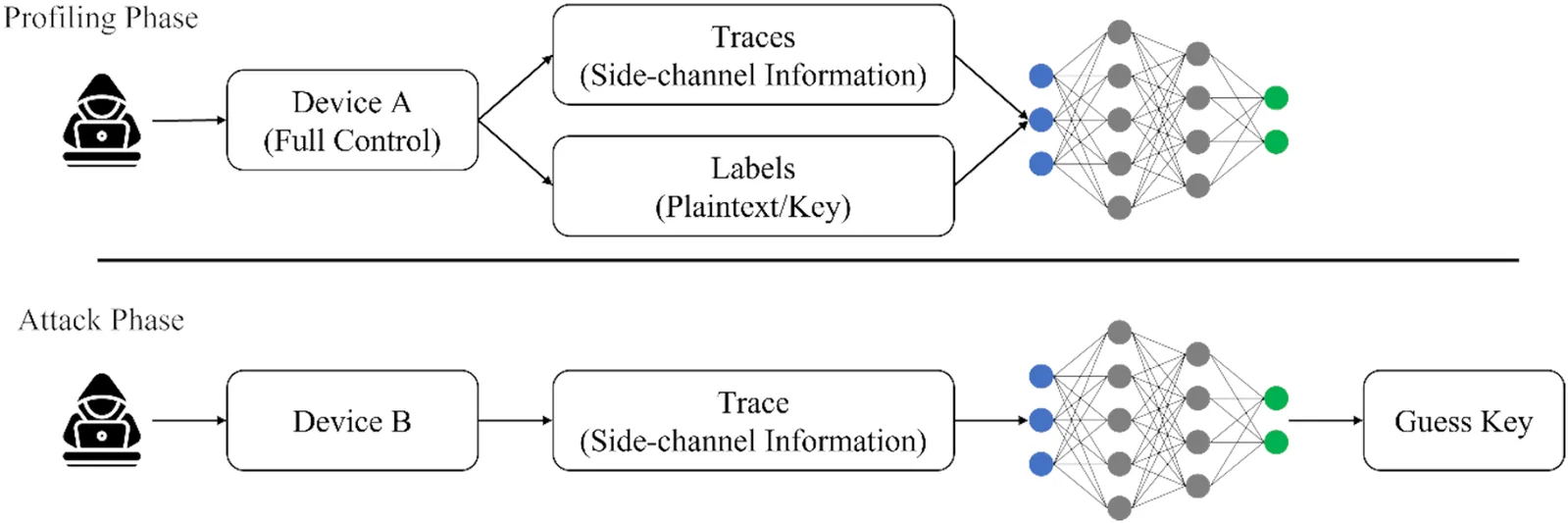
Deep-learning-based profiling attack workflow.

#### 2.2.3 SCA evaluation metrics.

To quantify attack effectiveness and defense efficacy in side-channel analysis, researchers commonly use a small set of metrics, most notably Key Rank, Guessing Entropy (GE), and Success Rate (SR) [12]. Historically, evaluations of attacks against primitives such as the Advanced Encryption Standard (AES) often focus on a specific intermediate value, such as the output of the first-round SubBytes operation, under the assumption of a fixed key and random plaintexts [6,13]. In that setting, GE and SR are natural metrics because they measure how quickly an attacker converges to the correct key or how many traces are needed to obtain a given confidence level.

The experimental setting of the present work differs in two important respects. First, the target for each trace is a random 32-byte message rather than a single fixed key. Second, we perform byte-wise classification, splitting each trace into 32 labeled segments so that the model predicts one byte per segment. Under these conditions, metrics that assume a fixed key (such as GE computed over key candidates) are not directly applicable. For this reason, we do not report GE in our evaluations.

Instead, we use two SR-based measures tailored to our problem. Let *T* denote the number of attack traces and let *B* = 32 be the number of bytes per trace. Denote by *y*_(*i*,*j*)_ the true value of byte *j* in trace *i*, and by $\;y_{\left(i,j\right)}^{\prime }$ the model’s prediction for that byte. We define the byte-level success rate for byte *j* as shown in formula (1).


$$\begin{array}{c} SR_{\text{Byte}}=\frac{1}{T\times B}\sum_{i=1}^{T}\sum_{j=1}^{B}I\left(y_{i,j}^{\prime }=y_{i,j}\right) \end{array}$$ (1)

Where I(·) is the indicator function. This metric reports the fraction of traces for which the model correctly predicts a particular byte.

We also define the number of SRs that can be completely cracked among all 32-byte random messages, as shown in formula (2).


$$\begin{array}{c} SR_{\text{Trace}}=\frac{1}{T}\sum_{i=1}^{T}1\left(\forall j\in \{0,1,...,31\}:y_{i,j}^{\prime }=y_{i,j}\right) \end{array}$$ (2)

The full-message SR is a stricter metric: it equals the proportion of traces for which every byte is predicted correctly. Both of these measures are straightforward to interpret and directly reflect attacker utility in our scenario. Byte-level SR reveals which message positions are more or less vulnerable to leakage and learning, while full-message SR captures the practical impact of an attack when complete message recovery is the adversary’s goal. We report both statistics in the experiments so that readers can assess per-byte vulnerability and the end-to-end success rate of single-trace attacks.

### 2.3 Multilayer perceptron

We use a multilayer perceptron (MLP) as the profiling model for DL-based SCA because each message byte is represented by a fixed-length point-of-interest (PoI) vector, making the task a 256-class classification problem well suited to an MLP. As shown in Fig 3, the model consists of an input layer, hidden layers, and a softmax output layer that produces the probability distribution over 256 byte classes. The MLP is trained using cross-entropy loss with the Adam optimizer, while batch normalization, dropout, and early stopping are applied to improve generalization under noise and device variation. Signal alignment and amplitude normalization are performed before training, and the detailed model settings are reported in the Methods section.

**Fig 3:**
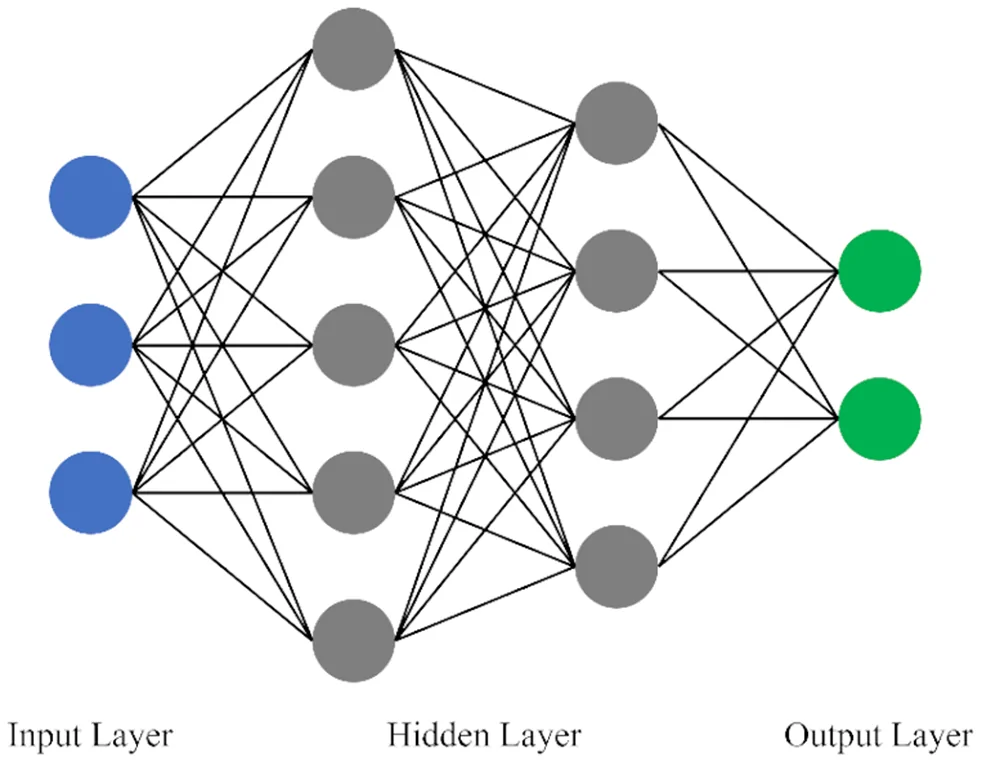
MLP architecture (illustrative diagram).

### 2.4 Low-pass filters

Low-pass filters are classical tools in signal processing, widely used to suppress high-frequency noise or to attenuate frequency components that may carry data-dependent features. In the context of SCA, they can directly influence the ability of DL models to extract meaningful leakage, thereby reducing the effectiveness of attacks. This study evaluates two common infinite impulse response (IIR) filters, namely Butterworth and Chebyshev filters, and systematically examines their impact on DL-SCA accuracy across different cutoff frequencies and filter orders. To minimize phase distortion, which could otherwise interfere with trace alignment, all filtering operations are applied using zero-phase implementations (e.g., forward–backward filtering). Details of the parameter settings, regularization, and discretization approaches are provided in the Methods section.

#### 2.4.1 Butterworth low-pass filter.

The Butterworth filter, first introduced in 1930 [14], remains one of the most widely adopted designs in both analog and digital applications. Its defining feature is the maximally flat response in the passband: within the frequency range below the cutoff frequency $\omega_{c}$, the response is smooth and free of ripples. This property makes the Butterworth filter particularly suitable for applications where preservation of signal fidelity within the passband is essential. The filter’s performance is governed by its order *n*, which determines how sharply the response falls off beyond $\omega_{c}$. Higher orders produce steeper roll-offs, but also increase implementation complexity. The transfer function of the Butterworth low-pass filter is given in formula (3), where *G* represents the frequency response, ω is the angular frequency (rad/s), $\omega_{c}$ is the cutoff frequency, and *n* is the filter order.


$$\begin{array}{c} G_{n}(\omega )=\left|H_{n}(j\omega )\right|=\frac{1}{\sqrt{1+{\left(\frac{\omega }{\omega_{c}}\right)}^{2n}}} \end{array}$$ (3)

#### 2.4.2 Chebyshev low-pass filter.

The Chebyshev low-pass filter is derived from the Chebyshev polynomial $T_{n}(\cdot )$ and is also implemented as an IIR filter. Unlike the Butterworth filter, it allows a controlled amount of ripple in either the passband or the stopband in exchange for a sharper transition. Two standard variants are commonly used:

Type I Chebyshev: permits a constant ripple in the passband while ensuring a monotonic decay in the stopband.
Type II (Inverse Chebyshev): maintains a flat passband while allowing ripple in the stopband.

In side-channel contexts, the steeper roll-off of the Chebyshev filter can more effectively suppress high-frequency leakage, which may otherwise assist DL models in distinguishing sensitive features. However, the allowance of ripple in the passband introduces minor amplitude fluctuations in the time domain, which may influence how well the model adapts to low-frequency components. In this study, we directly compare Chebyshev filtering against Butterworth filtering under multiple cutoff frequencies and filter orders to assess their relative ability to suppress learnable leakage. The transfer function is expressed in formula (4), where $T_{n}(\cdot )$ is the *n*th-order Chebyshev polynomial of the first kind, and other parameters follow the definitions in Equation (3).


$$\begin{array}{c} G_{n}(\omega )=\left|H_{n}(j\omega )\right|=\frac{1}{\sqrt{1+\epsilon^{2}T_{n}^{2}\left(\frac{\omega }{\omega_{c}}\right)}} \end{array}$$ (4)

## 3. Proposed method

This chapter outlines the experimental methodology developed to evaluate DL-SCA and filtering-based countermeasures on ML-KEM implementations in embedded systems. The methodology is split into six parts: defining the attack model and its assumptions, analyzing the target algorithm, collecting and measuring power traces, designing preprocessing procedures, constructing and training the deep learning model, and finally, implementing low-pass filter defenses. This step-by-step approach is meant to make sure we can repeat experiments and keep them accurate, while also giving us a way to judge and compare how PQC works in the future.

### 3.1 Attack model

The attack scenario considered in this study follows the standard assumptions of profiling-based side-channel analysis. The adversary is assumed to possess the following capabilities:

(a) Device control: The attacker has access to a microcontroller (MCU) that is the same as or works in the same way as the target device and can control all its computational processes.
(b) Input manipulation: The attacker can provide a large set of random inputs to the target device in order to repeatedly trigger the execution of the ML-KEM algorithm.
(c) Trace measurement: The attacker can accurately determine the start of the decryption process and capture power consumption traces during algorithm execution.

These assumptions reflect a realistic yet strong adversarial setting, where the combination of device control, input freedom, and precise measurement enables effective side-channel exploitation.

### 3.2 Algorithm analysis

The implementation analyzed in this work is based on the ML-KEM algorithm from the PQClean open-source library [15], which ensures correctness and portability across platforms. Particular emphasis is placed on the poly_frommsg() function, which transforms 32 bytes of random data into a 256-dimensional polynomial representation (see Algorithm 2 and Algorithm 3). In ML-KEM, each bit of the 32-byte message is mapped to one polynomial coefficient. To achieve constant-time execution, optimized C or assembly implementations usually avoid conditional branches such as if-else statements. Instead, they use bitwise operations to expand a single message bit into a 16-bit mask. Specifically, for each input bit parameter (b), the implementation derives the mask through a two’s-complement negation operation. When (b = 0), the resulting register value is 0x0000, whose Hamming weight is 0; when (b = 1), the register value becomes 0xFFFF, whose Hamming weight is 16.

Although this branchless implementation helps reduce timing-based leakage, it does not eliminate power-based leakage. A single message bit is amplified into either an all-zero or an all-one 16-bit value, producing the maximum possible Hamming-distance difference between the two cases. When the CPU bus or internal registers switch between these extreme states, the power consumption can change significantly, resulting in large data-dependent leakage peaks. Therefore, the constant-time coding style prevents control-flow leakage but does not fully prevent data-dependent power leakage. Prior research [10,16,17] has also demonstrated that this type of message-encoding operation remains vulnerable due to Hamming-weight variation. As summarized in Table 3., previous studies have reported single-trace or few-trace side-channel attacks against lattice-based KEMs such as NewHope, CRYSTALS-KYBER, SABER, FrodoKEM, and Kyber512. In contrast, this work focuses on ML-KEM implemented on an embedded STM32F415 platform and evaluates 32-byte message recovery under a DL-based profiling setting. Steffen et al. further identified this behavior in the CRYSTALS-Kyber message encoding function on ARM Cortex-M4 and explained that the two possible mask values in poly_frommsg() have the maximum Hamming distance, making the processed message bits distinguishable through the power side channel [18].

**Table 3:** Comparison of side-channel attacks on lattice-based KEMs.

Work	Algorithm	Attack target	Attack model	Traces
[10]	Kyber512	Secret key	Chosen-ciphertext EM SCA	4–960
[17]	NewHope	Message / shared secret	Passive power SCA	1
[18]	CRYSTALS-KYBER / SABER / FrodoKEM	Encoded message	Single-trace SCA	1
our	ML-KEM	32-byte message	DL profiling SCA	1

This large and consistent difference creates a strong relationship between the processed bit values and the observed power consumption, since microcontroller power consumption is affected by the Hamming weight or Hamming distance of the processed data. Consequently, these data-dependent variations can be exploited to reconstruct the encapsulated random message under a profiling-based side-channel setting. This interpretation is also consistent with the observation of Du *et al.*, who showed that data-dependent Hamming-weight behavior during storage operations in CRYSTALS-Kyber message decoding can leak message-related information and enable practical message and secret-key recovery [19]. Although their target is the message decoding function rather than poly_frommsg(), their results further support the underlying leakage mechanism that distinguishable Hamming-weight behavior in register or memory operations can lead to exploitable message leakage.

Because the recovered message participates in the subsequent decapsulation and shared-secret derivation flow, successful message recovery may compromise the confidentiality of the decapsulation process and materially weaken the implementation-level security guarantees. Therefore, we regard message recovery as a practically significant security breach, while noting that the exact downstream impact depends on the implementation context and the attacker’s degree of access to the surrounding protocol state.

**Algorithm 2. Polynomial Coefficient Extraction from Encapsulated Message**

**Input:** Polynomial *r*, Input Message in msg[32]

1:  **for** i=0...31 **do**

2:   **for** j=0...7 **do**

3:    r→coeffs[8·i+j]=0;

4:    cmov_int16(r→coeffs+8·i+j, (q+1)/2, (msg[i]≫j) & 1);

5:   **end for**

6:  **end for**

**Algorithm 3. Constant-Time Conditional Move (cmov_int16)**

**Input:** Pointer *r*, Input *v*, Condition bit *b*

1:  b=−b;

2:  *r⊕=b & ((*r)⊕v);

### 3.3 Power trace measurement

The initial step in performing a side-channel attack is to repeatedly execute the target algorithm in order to collect a sufficiently large set of power traces for analysis. In this study, power consumption measurements were taken from an STM32F415 microcontroller mounted on a ChipWhisperer CW308 board while running the ML-KEM algorithm. Because the CW-lite’s ADC is limited to capturing only 24,400 sampling points, traces obtained directly from this interface suffered from distortion. To obtain more accurate measurements, we employed an external PicoScope 5244B oscilloscope.

The STM32F415 integrates an internal low-dropout (LDO) linear regulator [20], which introduces considerable noise into the recorded traces. To mitigate this effect, we applied the built-in 20 MHz hardware low-pass filter of the PicoScope 5244B, effectively suppressing high-frequency noise [21–23]. Each captured power trace thus contained 455,000 sampling points, as illustrated in Fig 4.

**Fig 4:**
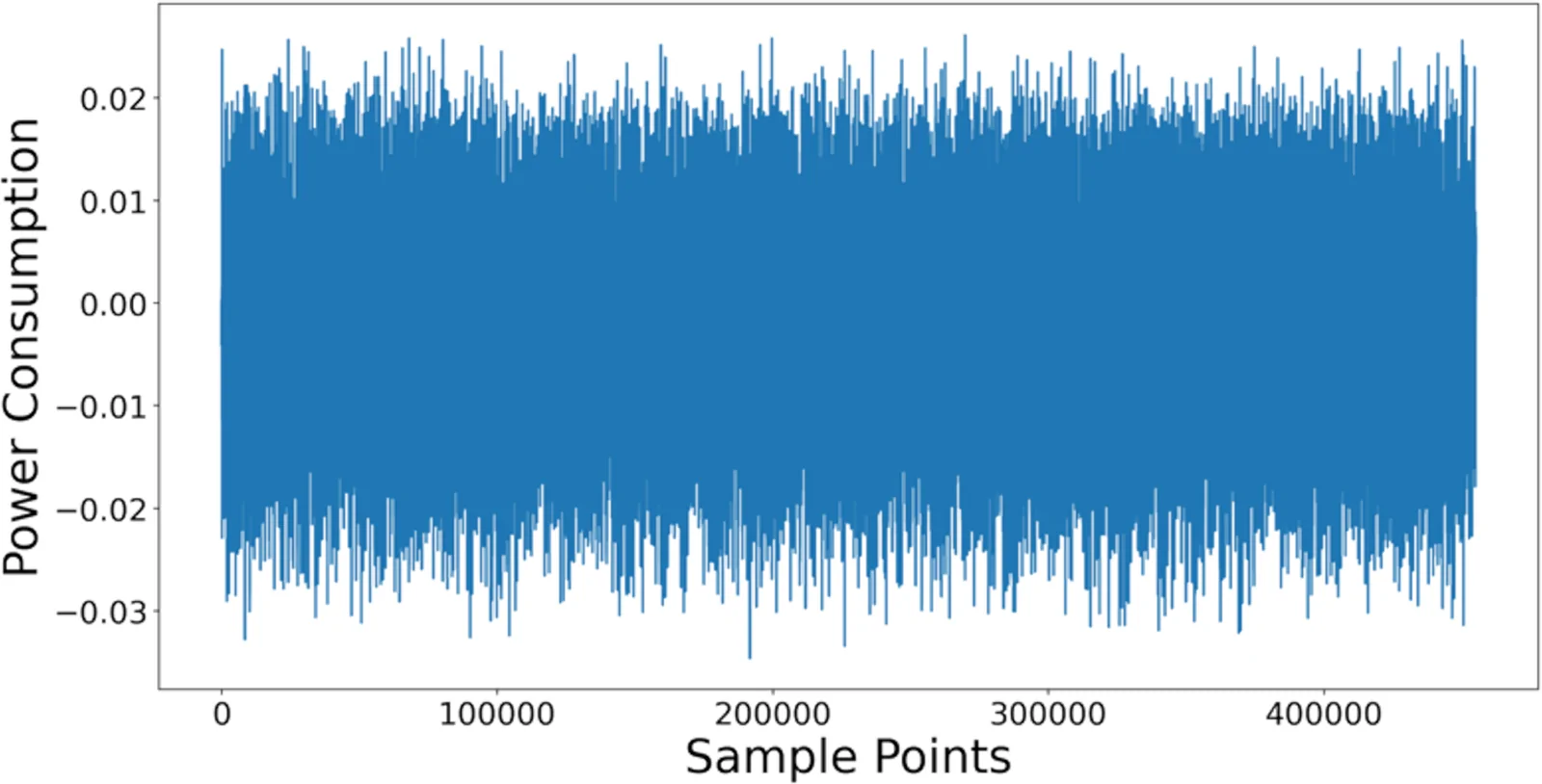
Example single power trace (raw).

To confirm the periodic behavior of the poly_frommsg() function, 2,000 random messages were supplied to the device. The averaged trace revealed 32 distinct peaks, each corresponding to an iteration of the algorithm’s outer loop, shown in Fig 5. Signal-to-noise ratio (SNR) analysis, calculated using formula (5), was then applied to identify points of leakage. The results demonstrated that each of the 256 message bits produced a recognizable peak, with the 21st bit showing the strongest leakage signature (Figs 6 and 7).


$$\begin{array}{c} SNR=\frac{Var\left(𝔼[L|X]\right)}{𝔼\left[Var(L|X)\right]} \end{array}$$ (5)

**Fig 5:**
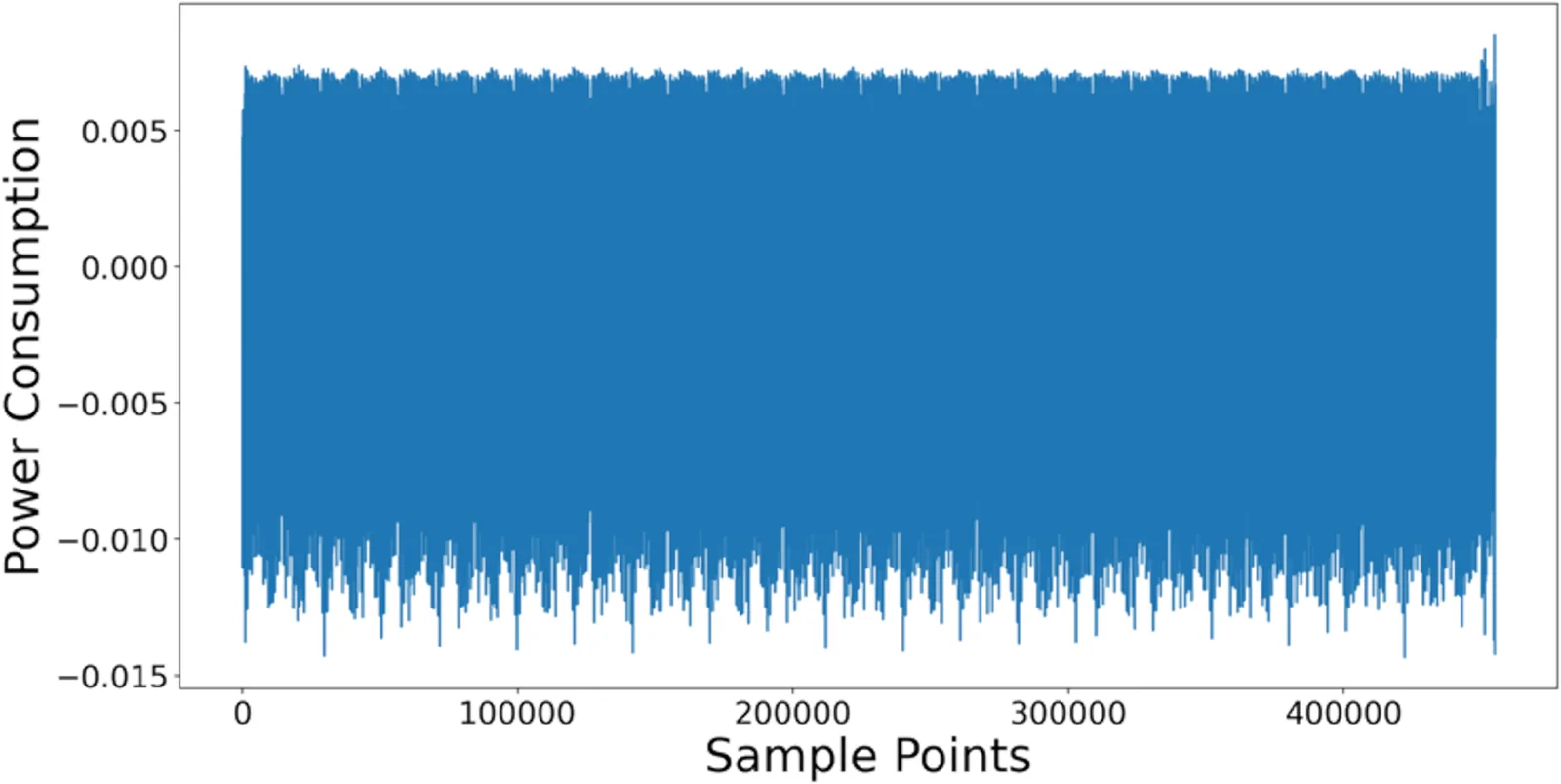
Mean trace over 2000 power measurements).

**Fig 6:**
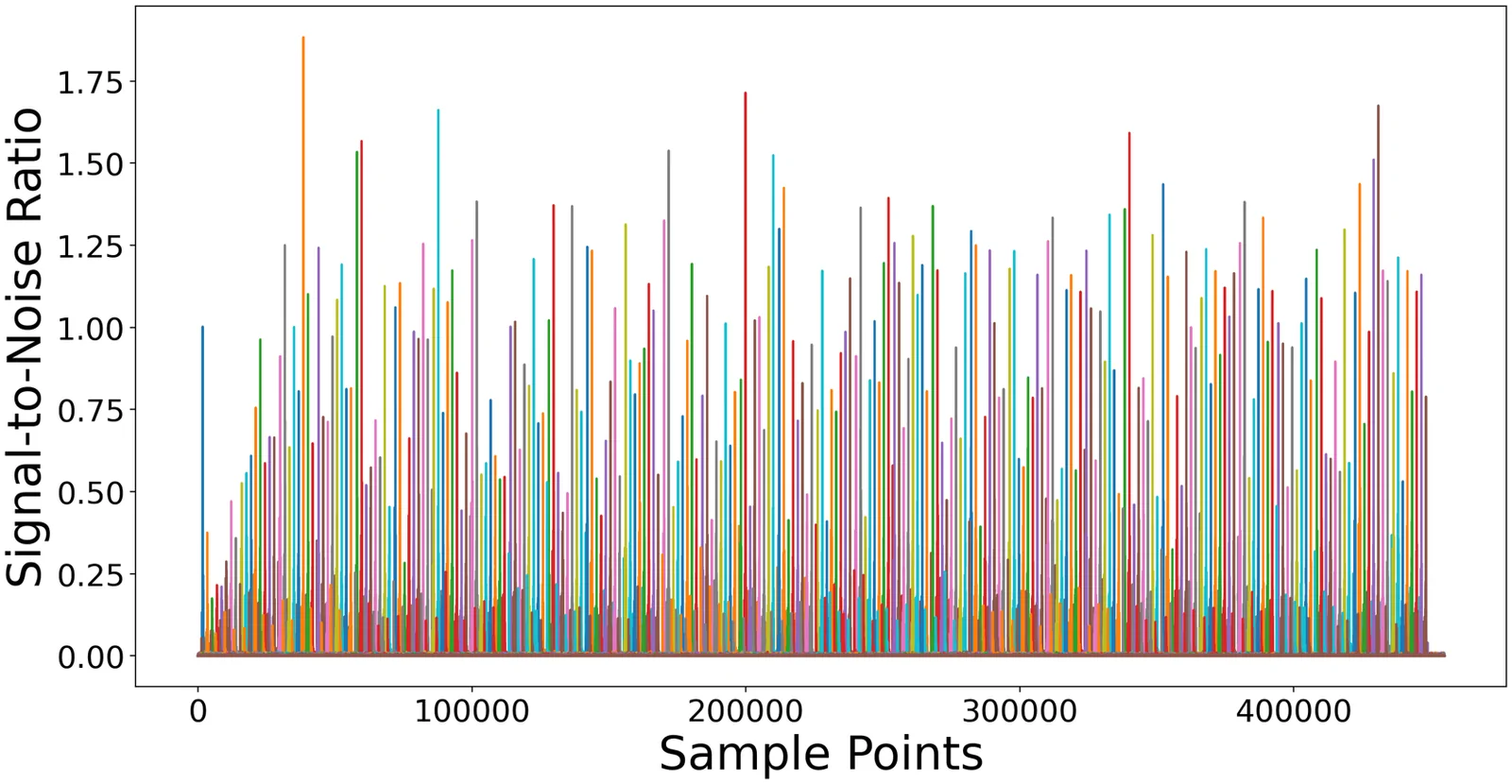
Signal-to-noise ratio across 256 bits.

**Fig 7:**
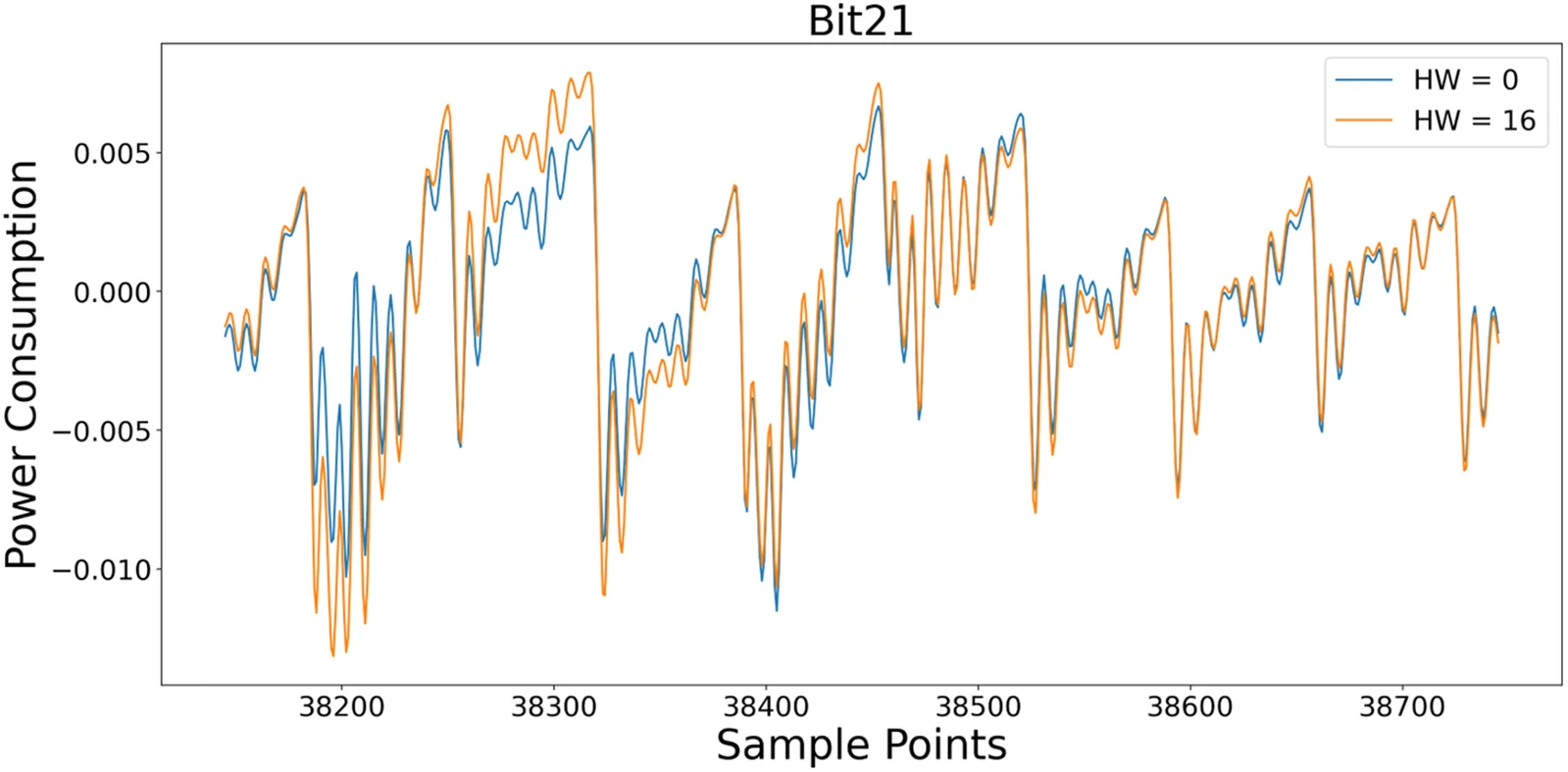
Clustering of bit 21 (leakage groups).

### 3.4 Power trace preprocessing

Each recorded power trace corresponds to a complete 32-byte package of random information. To reduce computational complexity and improve training efficiency, this study adopts a segmentation strategy at the byte level:

(a) Byte segmentation. Each trace is divided into individual byte segments, where the label corresponds to the actual value of that byte.
(b) PoI extraction. PoI windows are identified using SNR analysis. For each peak, 300 sampling points before and after the maximum value are retained as input features, as shown in Fig 8.
(c) Feature vector construction. The PoI windows corresponding to the 8 bits of a byte are concatenated to form a single input vector, as shown in Fig 9. This approach substantially reduces dimensionality, thereby lowering the computational cost of training.

**Fig 8:**
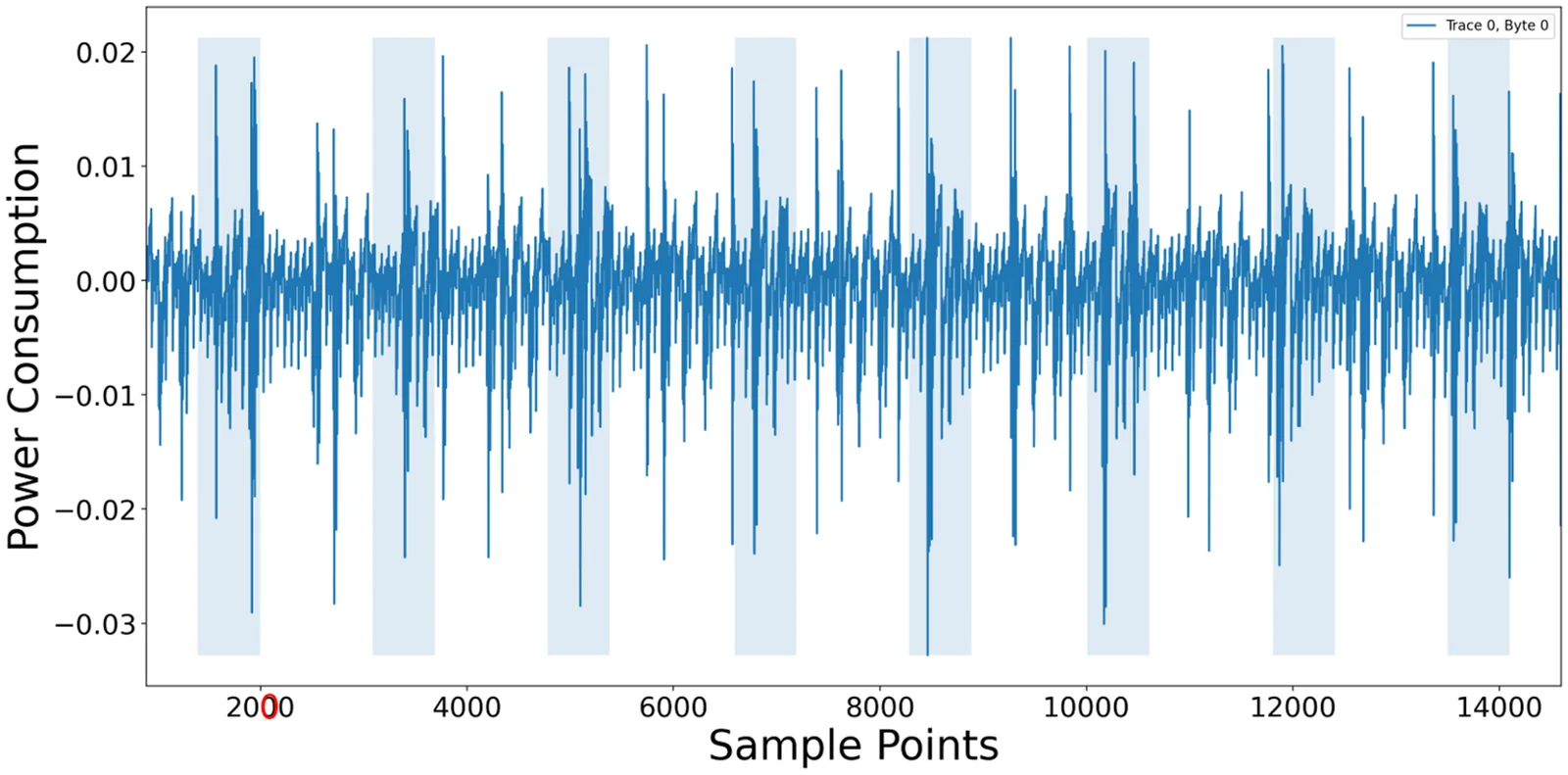
Clustering of bit 21 (leakage groups).

**Fig 9:**
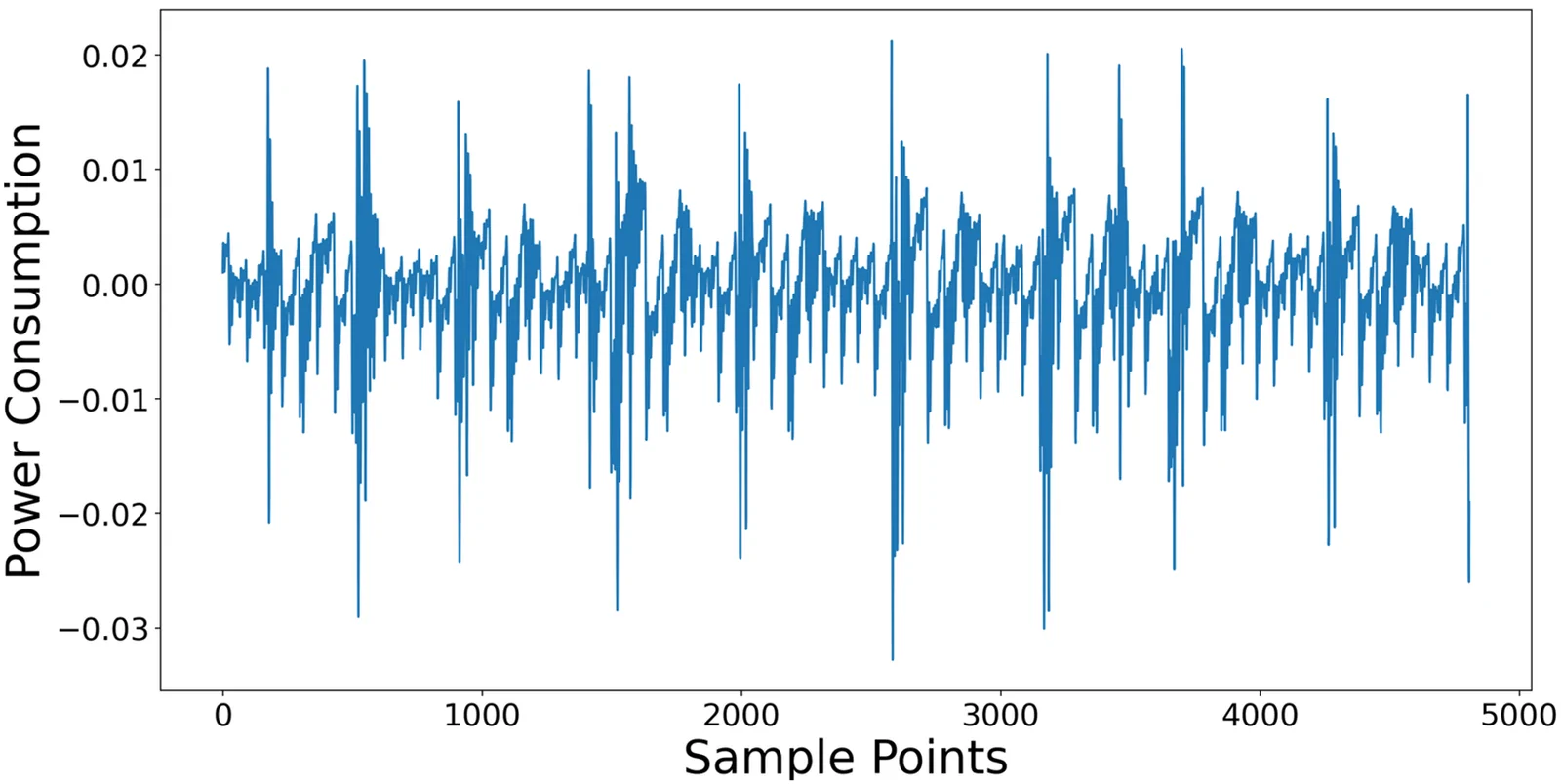
Concatenated PoI windows for byte 1.

### 3.5 Model construction and training

We selected a MLP as the profiling attack model because the leakage targeted in this work is localized and strongly data-dependent. In our attack setting, the points of interest are determined from the identified leakage region, and the input traces are sufficiently aligned around the operation of interest. Therefore, a fully connected MLP is suitable for learning the relationship between the selected trace samples and the corresponding message-bit labels without requiring a more complex feature-extraction architecture.

This choice is also consistent with prior deep-learning-based side-channel attacks on CRYSTALS-Kyber. Dubrova et al. [24] demonstrated message recovery attacks on masked CRYSTALS-Kyber implementations on ARM Cortex-M4 using neural-network-based profiling, and their model design includes an MLP architecture for recovering message bits from masked implementations. Wang et al. [25] further showed that profiled deep-learning-based power analysis can be used to recover shared keys and secret keys from first-, second-, and third-order masked Kyber implementations on ARM Cortex-M4. These studies indicate that neural-network-based profiling, including MLP-style models, is effective for Kyber-related message or key recovery scenarios.

Although convolutional neural networks (CNNs) are powerful in side-channel analysis, their main advantage is more pronounced when traces are affected by jitter, desynchronization, or when the points of interest are not precisely known. Cagli et al. [26] showed that CNNs can perform end-to-end profiling attacks without explicit trace realignment or precise POI selection, making them particularly suitable for misaligned traces and jitter-based countermeasures. In contrast, our experiments focus on a well-defined and localized leakage region. Therefore, using an MLP provides a simpler, more interpretable, and sufficiently expressive model for the evaluated attack scenario, while avoiding unnecessary architectural complexity.

The network architecture was designed as follows:

(a) Input layer. The input dimension was 4,808, obtained by concatenating the selected PoI windows.
(b) Hidden layers. Two hidden layers were used, with 2,048 and 512 neurons in each. To classify each byte value, we adopted a MLP with an input dimension of 4,808, followed by two hidden layers of 2,048 and 512 neurons, and a 256-way softmax output layer. Batch normalization and dropout were applied after each hidden layer to improve generalization and reduce overfitting.
(c) Output layer. The output dimension was set to 256, corresponding to the full range of possible values for a single byte.

Hyperparameter selection was performed through randomized search over the candidate settings listed in Table 4, including the number of layers, hidden-layer width, activation function, loss function, learning rate, batch size, and training epochs. The final configuration used ReLU activations in the hidden layers, cross-entropy loss, the Adam optimizer with a learning rate of $1\times 10^{-4}$, a batch size of 512, and a maximum of 150 epochs.The final model architecture and its corresponding number of parameters are presented in Table 5.

**Table 4:** Model hyperparameters.

Hyperparameter	Attempted Value	Optimal Value
Number of Layers	[3, 4]	4
Outputs Size of Layers	[512, 1024, 2048]	[4808, 2048, 512, 256]
Activation Function	[ReLU, GeLU]	ReLU (Other Layer), Softmax (Output Layer)
Loss Function	[Cross-entropy, MSE]	Cross-entropy
Dropout Rate	[0.1, 0.3, 0.5]	0.5
Optimizer	[Adam, RMSprop]	Adam
Batch Size	[64, 128, 256, 512]	512
Epochs	[100, 150, 300, 1000]	150
Learning Rate	[0.001, 0.005, 0.0001, 0.0005]	0.0001

**Table 5:** MLP model architecture.

Layer	Output Shape	Parameter #
dense	(None, 2048)	9,846,784
batch_normalization	(None, 2048)	8,192
activation	(None, 2048)	0
dropout	(None, 2048)	0
dense_1	(None, 512)	1,048,576
batch_normalization_1	(None, 512)	2,048
activation_1	(None, 512)	0
dropout_1	(None, 512)	0
dense_2	(None, 256)	131,328

This model was selected because it provided the best validation performance among the tested candidates while maintaining a relatively lightweight architecture for byte-wise inference on fixed-length PoI feature vectors.

Before training, traces were aligned using the profiling-device reference positions, normalized in amplitude, and segmented into byte-wise PoI feature vectors. The profiling and evaluation datasets were separated at the trace level to avoid leakage between training and validation samples. Future releases of the artifact should report the random seed, exact data split, and implementation scripts to further improve reproducibility.

This configuration consistently achieved attack accuracies exceeding 99% with a single power trace, underscoring both the effectiveness of the MLP and the susceptibility of ML-KEM implementations to deep learning-based side-channel analysis.

### 3.6 Defense strategy

This study introduces a defense approach based on low-pass filtering to suppress high-frequency components in power traces, thereby limiting the ability of deep learning models to extract meaningful features. Two classical infinite impulse response (IIR) filters were examined:

(a) Butterworth low-pass filter. This filter is designed to have a flat passband response, which means that the signal is as close to a pure sine wave as possible. It has a 1 MHz cutoff frequency and a 5th-order design, which means that it can be used over a wide range of frequencies without distorting the signa, as shown in Figs 10 and 11.
(b) Chebyshev low-pass filter. Configured with a passband ripple of 1 dB, this filter achieves a sharper transition between passband and stopband compared to the Butterworth design, though at the cost of introducing controlled ripple in the passband, as shown in Figs 12 and 13.

**Fig 10:**
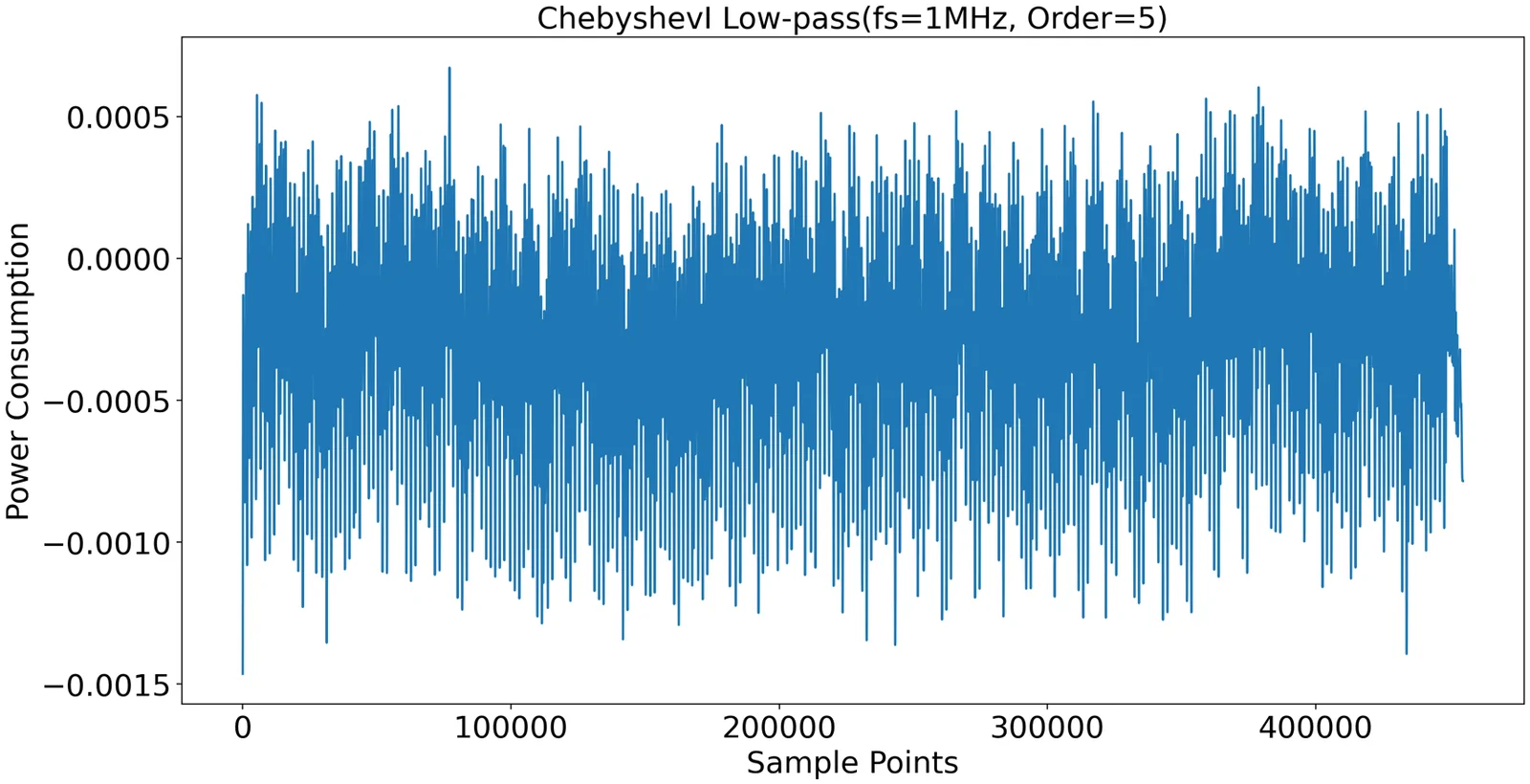
Single power trace after Butterworth LPF.

**Fig 11:**
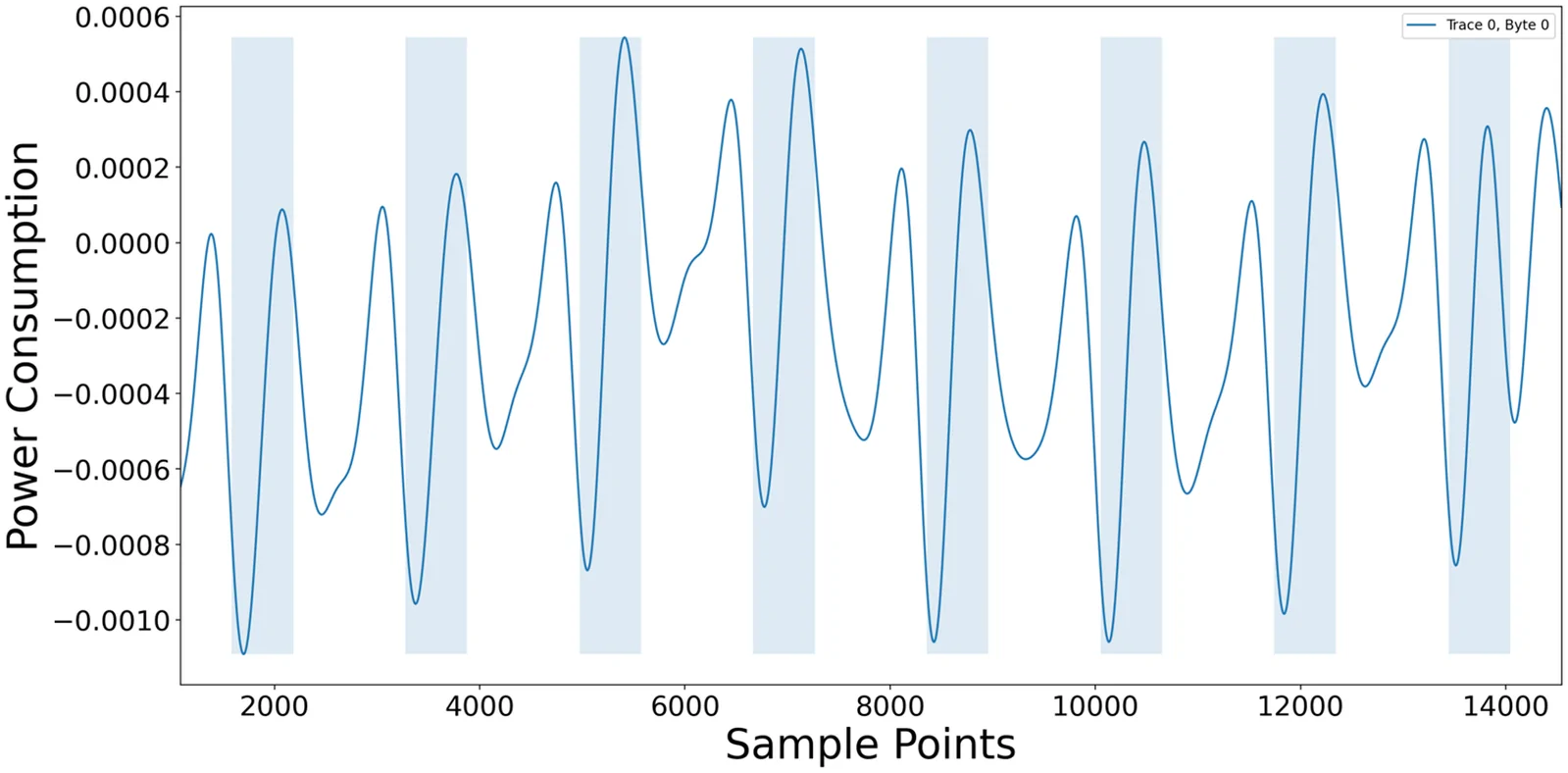
PoI window after Butterworth LPF.

**Fig 12:**
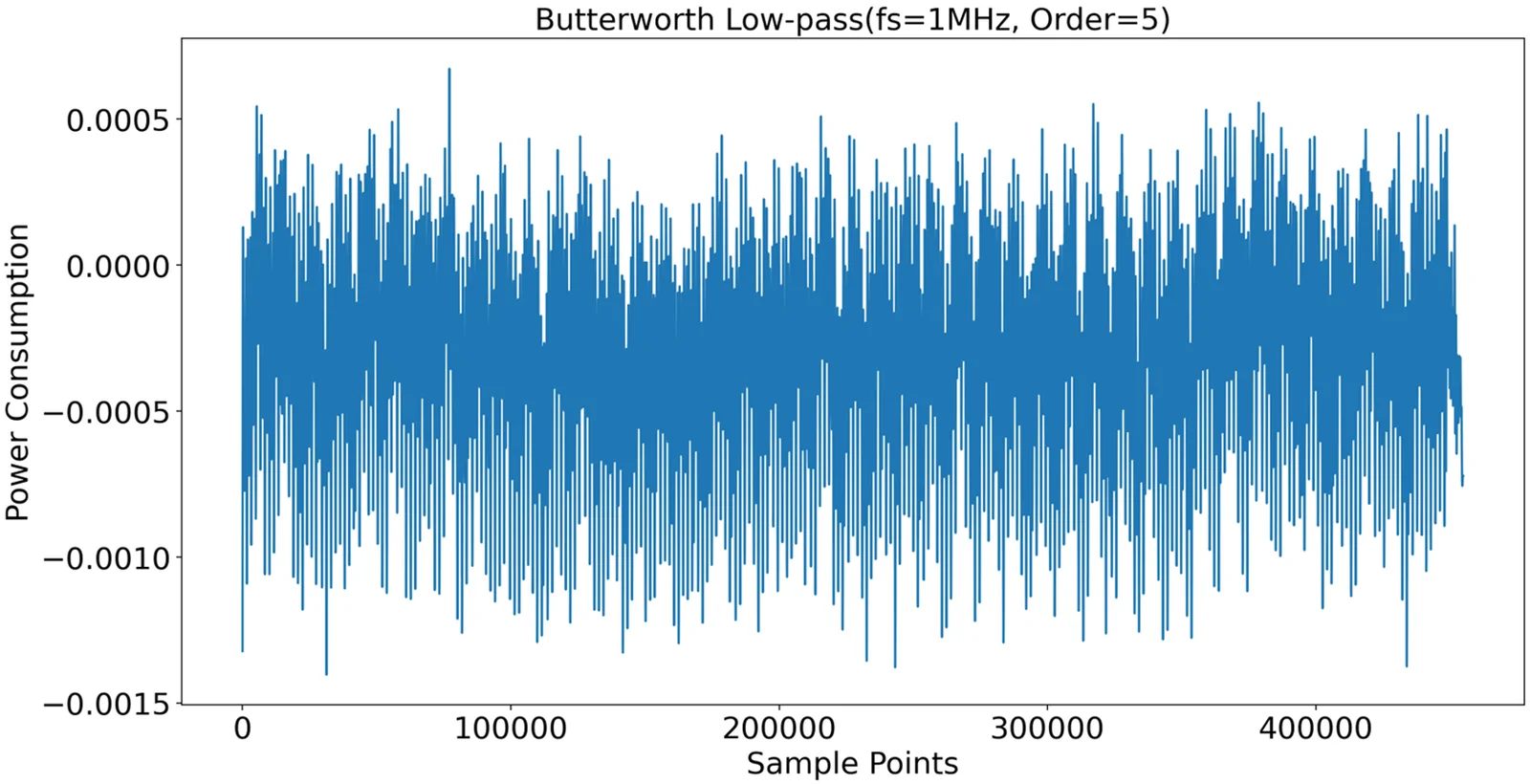
Single power trace after Chebyshev Type-I LPF.

**Fig 13:**
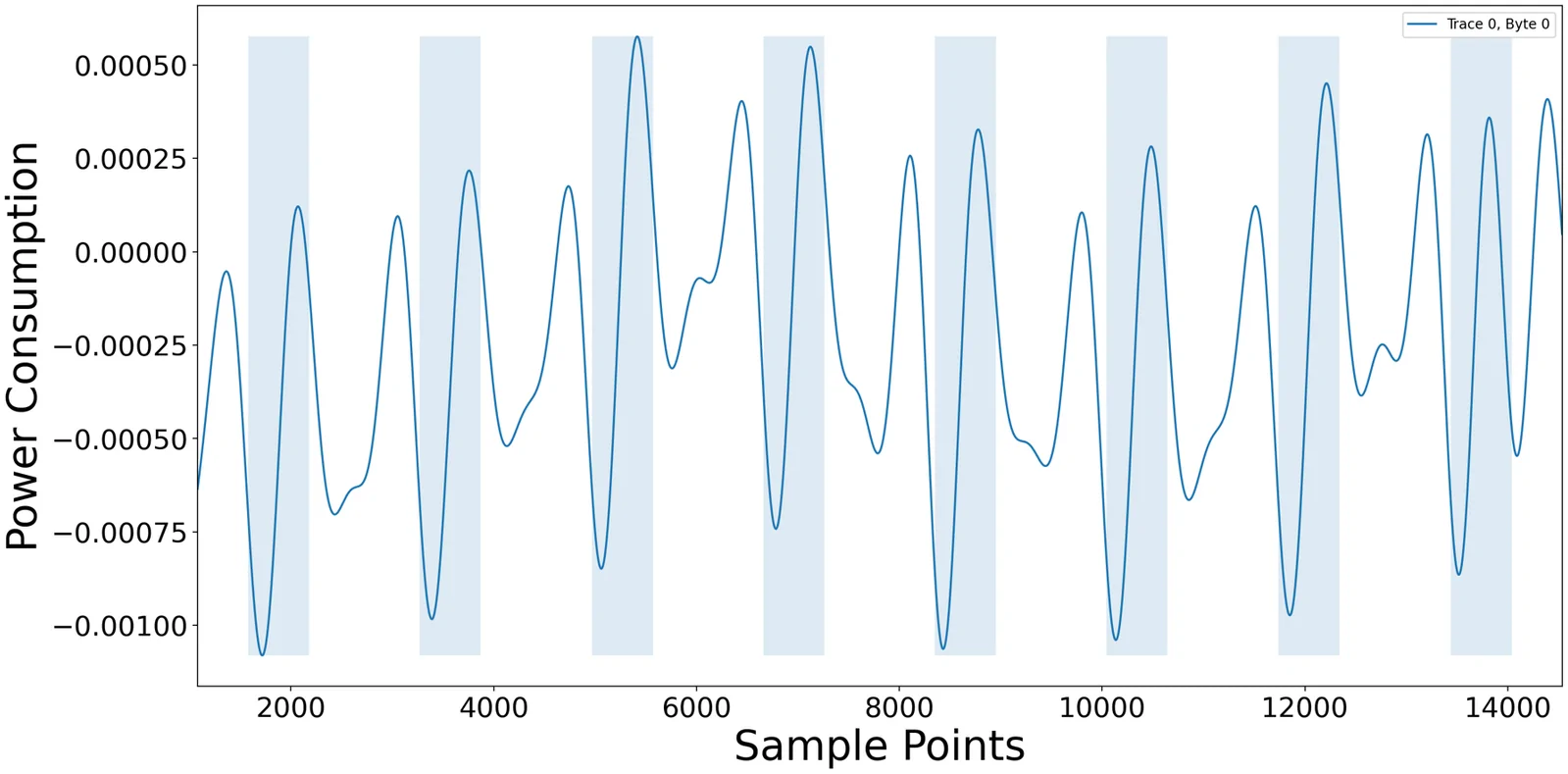
PoI window after Chebyshev Type-I LPF.

Both filters were applied to the collected power traces, after which the data underwent the same preprocessing pipeline and classification with the MLP model described earlier. Experiments show that filtering reduced the success rate of attacks from nearly 100% to between 9% and 65%, depending on the configuration. These results should be interpreted as a controlled sensitivity analysis rather than as a claim that offline digital filtering alone can defend a deployed device against a realistic adversary. Since an attacker with access to raw traces can apply arbitrary post-processing, the present experiments do not restrict the attacker’s capabilities. Instead, they reveal that the learned classifier depends strongly on specific spectral components, especially under higher-order filtering and lower cutoff frequencies.

From a defensive perspective, this observation is still valuable: it suggests that practical protections should focus on suppressing, randomizing, or decorrelating those exploitable components in the physical leakage path. Candidate directions include masking, shuffling, temporal desynchronization, and board-level or on-chip power/EM conditioning, all of which require separate implementation and overhead evaluation.

## 4. Experimental results

This chapter presents the experimental findings that validate the feasibility of deep-learning side-channel attacks against ML-KEM implementations and evaluate the effectiveness of filtering-based countermeasures. Section Experimental Architecture describes the hardware setup and overall experimental architecture. Section Power Trace Quality Analysis compares model performance when trained on equal-sized trace sets, while Section Selection of Training Set Size examines how varying the number of training traces affects results. Section Training Time Comparison reports training time and resource comparisons between CPU and GPU-based training. Section Attack Results and Cross-Device Generalization summarizes attack success rates, including cross-device evaluations on multiple MCUs. Finally, Section Effectiveness of Defense Mechanisms assesses the mitigation impact of Butterworth and Chebyshev low-pass filtering under different parameter settings.

### 4.1 Experimental architecture

The experimental platform used in this work is summarized in Table 6 and consists of four main components: a control PC, a GPU computing server, an oscilloscope, and the target microcontroller (STM32F415). The control host runs Ubuntu 22.04.4 LTS and orchestrates the experiment by delivering randomized 32-byte messages to the ChipWhisperer CW308 target board. Meanwhile, a PicoScope 5244B oscilloscope records the MCU’s power consumption during ML-KEM execution. To obtain finer-grained power trace measurements, the traces were sampled at 2 ns intervals, corresponding to 500 MS/s. Based on the Nyquist sampling theorem, this sampling rate can observe frequency components up to 250 MHz. This oversampling setting helps retain high-frequency leakage and switching-related noise generated during ML-KEM execution.This sampling setting is consistent with prior side-channel evaluations of lattice-based cryptographic implementations. Specifically, Cui et al. used a 2 ns sampling interval when evaluating side-channel-resistant NTT implementations, while Reparaz et al. sampled the power consumption of a masked Ring-LWE implementation at 500 MS/s [27,28]. The raw waveforms were streamed back to the control PC for storage and offline processing.

**Table 6:** Hardware and instrumentation.

Hardware Component		Specifications
Controller PC (VM)	OS	Ubuntu 22.04.4 LTS
	CPU	Intel(R) Core(TM) i7-12700
	RAM	20GB DDR5 4800MHz
	Interface	Ethernet, USB
GPU Computing Server	OS	Ubuntu 22.04.3 LTS
	CPU	Intel(R) Core(TM) i7-13700
	RAM	32GB DDR4 3200MHz × 2
	GPU	NVIDIA GeForce RTX 3090 Ti GDDR6X 24GB
	Interface	Ethernet
Oscilloscope	Device	PicoScope 5244B
	Interval	2 ns
	Duration	910 μs
	VRange	200 mV
	Resolution	12 bits
	Interface	USB
Target	MCU	STM32F415
	Clock Rate	7.37 MHz
	Optimization	-O3
	Interface	USB

Because training deep learning models is computationally intensive, the collected datasets were transferred to a dedicated GPU server for model development and evaluation. All training and inference experiments were implemented in TensorFlow 2.16.1 with CUDA 12.3 acceleration to ensure reproducible performance measurements. The complete end-to-end measurement and analysis pipeline is illustrated in Fig 14. The architecture and measurement procedures were designed to maximize repeatability and to provide clear conditions for comparing attack and defense configurations Fig 15.

**Fig 14:**
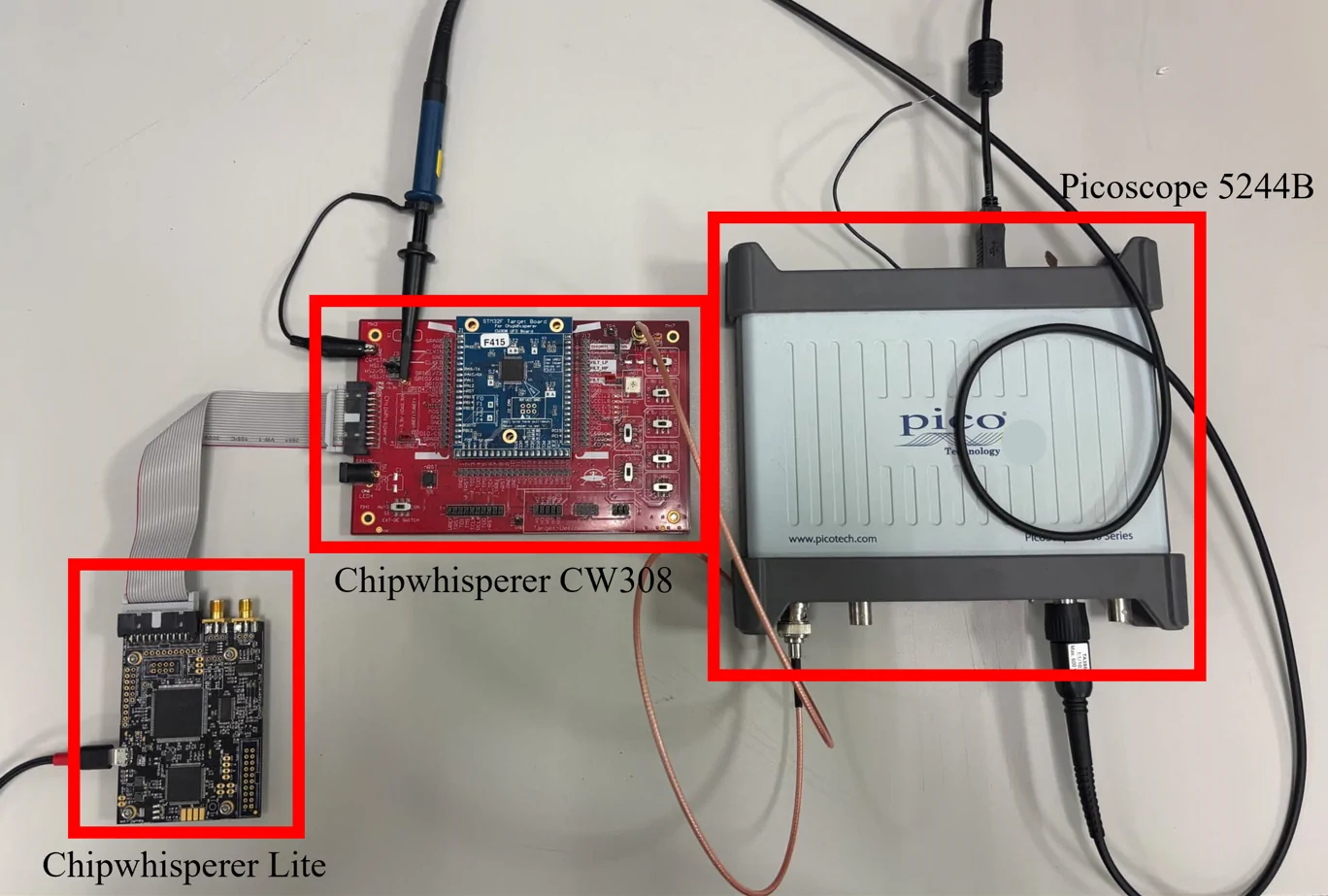
Overall side-channel attack architecture.

**Fig 15:**
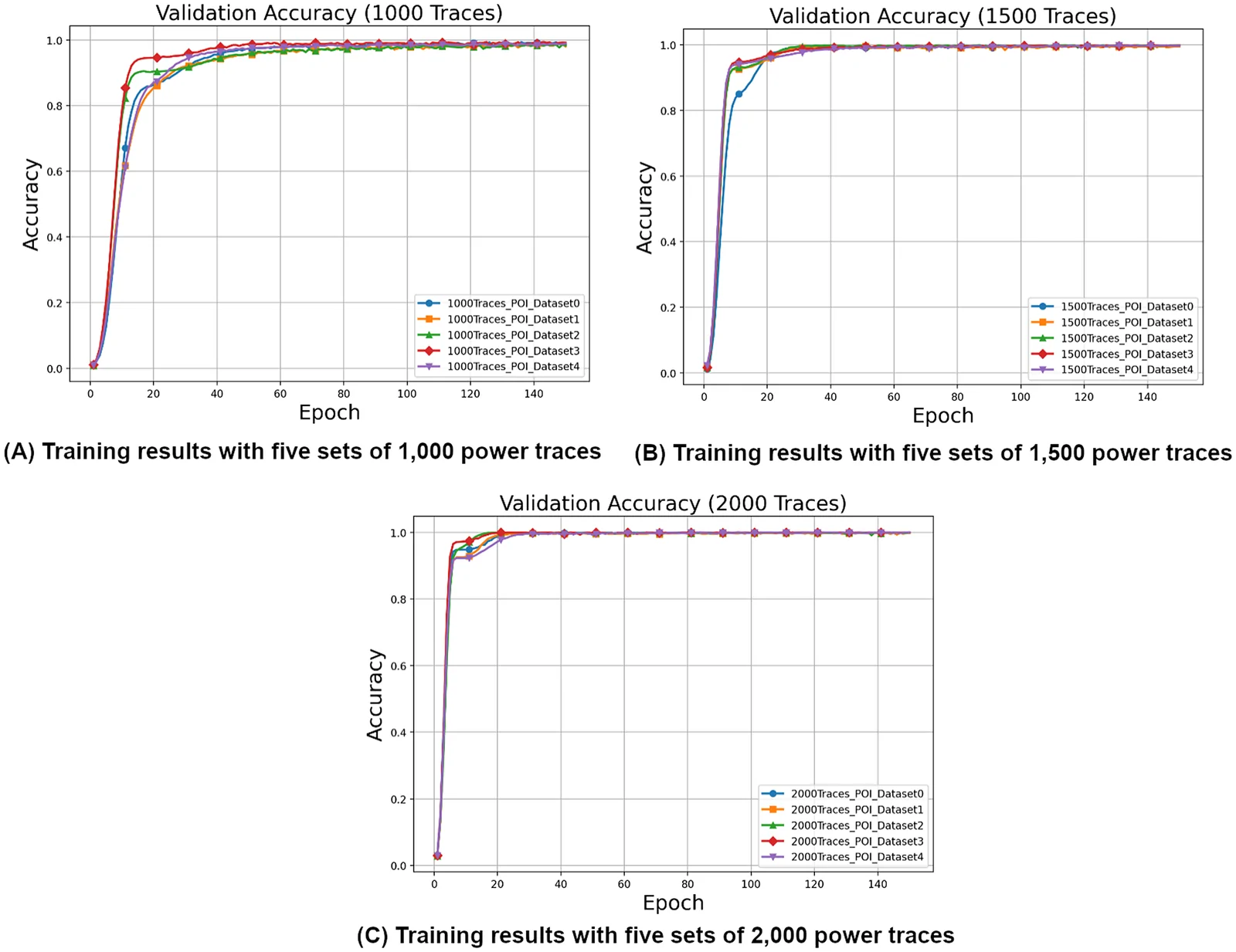
Deep-learning-based profiling attack workflow.

### 4.2 Power trace quality analysis

To ensure both representativeness and robustness, this study collected 500 traces for attack evaluation and three training datasets consisting of 1,000, 1,500, and 2,000 traces, respectively. The experiments show a clear link between how well the training works and the SNR: datasets with higher SNR values not only work out faster, but also are more accurate in predicting future results. Fig 16 and Table 7 show the results. As shown in Table 6 and Fig 15, increasing the profiling set from 1,000–2,000 traces consistently improves validation performance across the five independently constructed datasets. Although even 1,000 traces already yield high byte-level accuracy, the 2,000 trace setting produces the most stable and near-saturated results, with validation accuracy ranging from 99.77% to 100%.

**Table 7:** STM32F415: Dataset training results.

Number of Traces	Dataset0	Dataset1	Dataset2	Dataset3	Dataset4
	(%)				
1000 Traces	99.41	98.87	98.64	**99.45**	98.94
1500 Traces	99.61	99.61	99.96	99.90	**99.91**
2000 Traces	99.98	99.77	99.93	**100**	99.94

**Fig 16:**
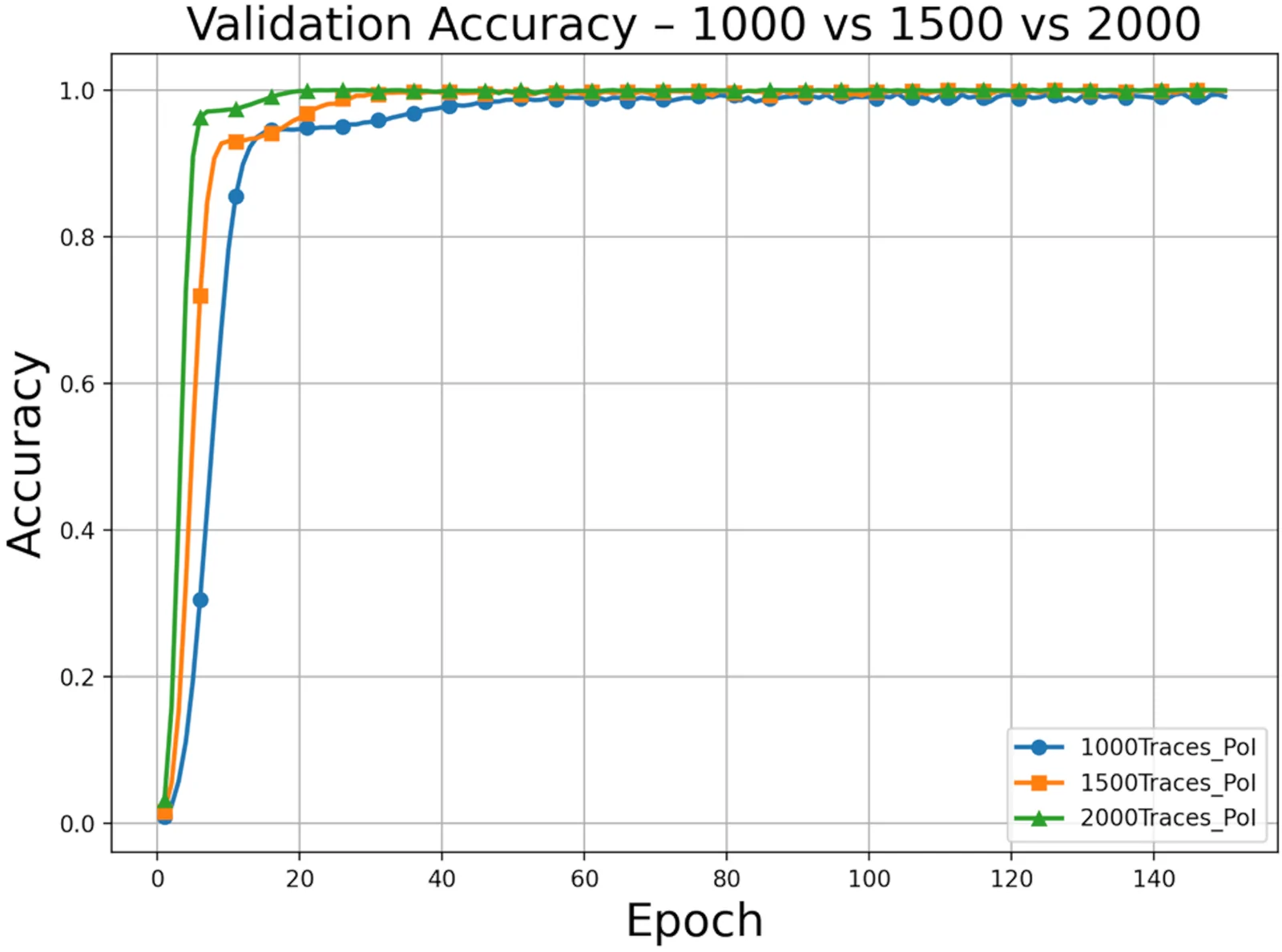
Overall side-channel attack architecture.

### 4.3 Selection of training set size

Fig 17 illustrates the effect of training set size on model performance. For this reason, the remaining experiments adopt 2,000 profiling traces as the default setting. We interpret this choice not merely as maximizing accuracy, but as reducing the sensitivity of the trained model to dataset-level fluctuation and improving generalization to previously unseen traces. Although a relatively small number of traces can already mount an effective attack, the larger dataset provides stronger generalization, reducing the risk of overfitting and maintaining accuracy on previously unseen traces. For this reason, the subsequent experiments are based on models trained with 2,000 traces.

**Fig 17:**
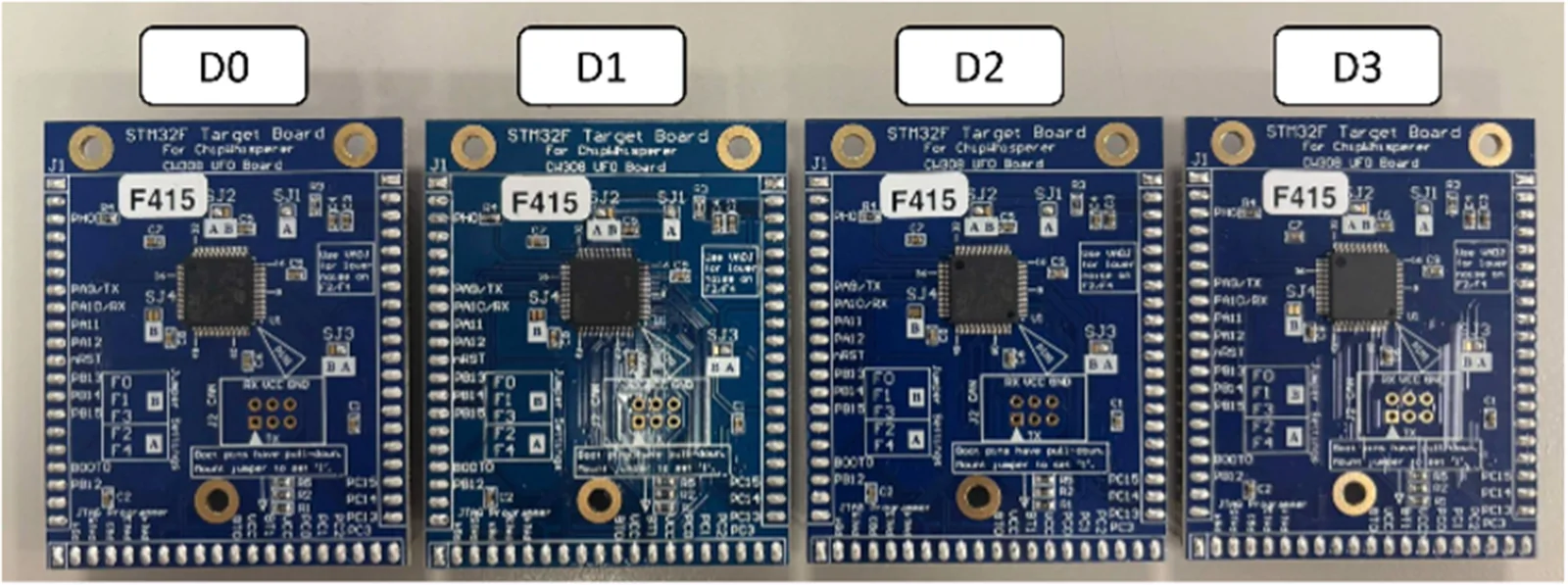
Device labeling map for cross-device experiments.

### 4.4 Training time comparison

To assess the practicality of the attack under different computational environments, we compared model training times on CPU and GPU platforms in Table 8. The GPU accelerated training by approximately an order of magnitude, but even on CPU-only systems, training remained feasible within a reasonable time budget. This indicates that the proposed the attack method has a relatively low computational barrier to entry. We also compared byte-wise and bit-wise recovery strategies. Furthermore, this study compared the differences between byte-based and bit-based restoration: while bit-wise methods can reduce model complexity and training time, byte-wise methods are more suitable for restoring the complete message. The choice of these two methods depends on the attack scenario.

**Table 8:** Training time comparison on STM32F415 datasets (minutes).

Number of Traces	Platform	Dataset0	Dataset1	Dataset2	Dataset3	Dataset4
1000 Traces	CPU	12.31	12.3	12.22	12.25	12.28
	GPU	1.43	1.37	1.38	1.34	1.37
1500 Traces	CPU	18.57	18.28	18.26	18.25	18.64
	GPU	1.86	1.8	1.79	1.84	1.84
2000 Traces	CPU	24.41	24.31	24.23	24.29	24.48
	GPU	2.35	2.28	2.26	2.28	2.32

### 4.5 Attack results and cross-device generalization

The primary attack objective is to recover the 32-byte encapsulated random message; because this message is unknown a priori, the PoI cannot be located directly from an attack trace. To overcome this limitation, we collected 2,000 profiling traces on a profiling device (denoted D0) and used that dataset to identify PoI windows and to train the classifier. All attack traces were then segmented according to the profiling-derived PoIs and submitted to the trained model for prediction. To assess how well the attack transfers across hardware instances [29–31], we evaluated the trained model on three additional STM32F415 microcontrollers (D1, D2, and D3). For each device, we recorded 500 traces. Although these units share the same model number, small process and manufacturing variations produce measurable differences in their power signatures shown in Fig 17. These differences can degrade transferability; nonetheless, a single model trained on D0 was applied to each target device without per-device retraining. We report two performance measures defined in Section 2.2.3: the byte-level success rate (fraction of correctly predicted bytes) and the full-message success rate (fraction of traces for which all 32 bytes are correct). Table 9 shows that byte-level recovery remains above 99% on all four devices, including the three non-profiling targets. However, the full-message success rate exhibits substantially larger variation: 99.4% on D0, 75.6% on D1, 97.2% on D2, and 81.4% on D3. This discrepancy highlights an important practical point: even when per-byte classification is highly accurate, small per-byte error probabilities accumulate rapidly when the adversary’s goal is complete 32-byte recovery.

**Table 9:** Cross-device attack results.

Device	Type	Correct Count	Total Count	Success Rate
D0 (Profiling)	Byte	15997	16000	99.9813%
	Message	497	500	99.4%
D1	Byte	15852	16000	99.0750%
	Message	378	500	75.6%
D2	Byte	15986	16000	99.9125%
	Message	486	500	97.2%
D3	Byte	15894	16000	99.3375%
	Message	407	500	81.4000%

The variation in full-message success rate also suggests that nominally identical devices still exhibit measurable domain shift, likely arising from manufacturing variability, analog front-end differences, clocking conditions, or trace-alignment sensitivity. These findings strengthen the practical relevance of the attack while also indicating that cross-device profiling should be evaluated using both byte-level and message-level metrics rather than either one alone.

Since the main focus of this work is to evaluate the effectiveness of the proposed defense rather than to optimize cross-device transferability, we do not further tune the attack model for each target device. Nevertheless, the performance gap between D1 and D2 provides useful insight into the practical limitations of single-device profiling. The lower full-message success rate on D1 is likely caused by a larger leakage-distribution mismatch with respect to the profiling device D0. Such mismatch may arise from physical-level differences, including semiconductor manufacturing parameter variations and differences in the power delivery network, as well as analog front-end differences, clocking behavior, or trace-alignment sensitivity. Since full-message recovery requires all 32 bytes to be correctly predicted in the same trace, even a small increase in byte-level errors can lead to a noticeable drop in full-message success rate.

This observation does not change the main conclusion of this work, but it highlights that cross-device profiling should be interpreted carefully. A possible extension is to construct the profiling dataset from multiple boards so that the model can learn a broader range of device-specific leakage distributions and reduce cross-device performance variation.

### 4.6 Effectiveness of defense mechanisms

This section reports a frequency-band sensitivity analysis using low-pass filtering applied to the recorded power traces. In a realistic profiling setting, an adversary who can capture raw traces can perform arbitrary offline signal processing; therefore, digitally filtering recorded traces should not be interpreted as constraining a real attacker. Instead, we use controlled filtering to quantify how strongly the deep-learning classifier relies on different spectral components of the leakage.

From an engineering perspective, these results help explain where the most exploitable leakage energy resides and can guide implementable mitigation choices. If the attack predominantly relies on higher-frequency components, practical defenses may prioritize measures that reduce or randomize high-frequency leakage (e.g., masking/shuffling, desynchronization, and board-level/on-chip power or EM conditioning in the physical acquisition path).

Deployment and overhead. The filtering experiments in this paper are performed offline on the control PC as part of the analysis pipeline; thus, they introduce no runtime overhead to the ML-KEM implementation. Any practical mitigation derived from these findings must be evaluated separately in terms of hardware feasibility, latency, energy, and functional impact on the embedded device.To make this analysis reproducible and to clarify the experimental basis of the selected frequency range, the rationale and procedure for choosing the filter cutoff frequencies are described in Section 4.7.

#### 4.6.1 Butterworth low-pass filter.

Table 10 presents the results of applying a Butterworth low-pass filter to 2,000 training traces, with different cutoff frequencies and filter orders. The experiments show that, for a fixed cutoff frequency, the filter order is very important for how accurate the model is. For example, with a cutoff at 1 MHz, a first-order filter still achieves 100% validation accuracy. When you’re dealing with a low order, the filter mostly gets rid of background noise. This makes the SNR better and helps the deep learning model work more efficiently. Fig 18 shows that when the order is raised to five, the accuracy drops a lot to 64.9%. At orders of 20 and 30, the results fall further, to 59.2% and 58.0% respectively. This pattern shows that higher-order filters create steeper attenuation at the edge of the passband, cutting off high-frequency leakage signals that are strongly tied to the random message. While this reduces the model’s ability to distinguish features, the accuracy drop beyond order 20 is less than 1%, suggesting that additional gains are minimal and the benefit of very high orders quickly levels off. A similar effect can be seen when the cutoff frequency is reduced while the filter order is fixed. The implementation of a fifth-order filter has been demonstrated to result in a consistent decline in validation accuracy, from 64.9% to 23.4%, with intermediate results of 62.5%, 56.7%, 48.4%, and 36.5%, as the cutoff is decreased from 1 MHz to 500 kHz. The findings demonstrate that decreasing the cutoff frequency results in a greater elimination of high-frequency leakage, thereby hindering the model’s capacity to extract meaningful features. Taken together, the results show that using a higher-order filter with a lower cutoff frequency can effectively weaken deep learning-based side-channel attacks. However, pushing the order too high provides little additional protection while adding extra implementation cost. In practice, a balance between stronger security and manageable system overhead is the most effective choice.

**Table 10:** Butterworth LPF: Validation accuracy by cutoff frequency and filter order.

Cutoff Frequency	1	5	10	15	20	25	30
	%						
1 MHz	100	64.9	60.8	59.8	59.2	59	58.3
900 kHz	100	62.5	57.6	56.7	55.5	55.2	54.5
800 kHz	100	56.7	51.3	48.4	47.3	46.6	45.8
700 kHz	100	48.4	41.3	38.4	37.1	36.5	35.8
600 kHz	99.9	36.5	28.6	26.3	25.4	24.5	24.3
500 kHz	99.9	23.4	15.4	12.8	11.9	11	10.9

**Fig 18:**
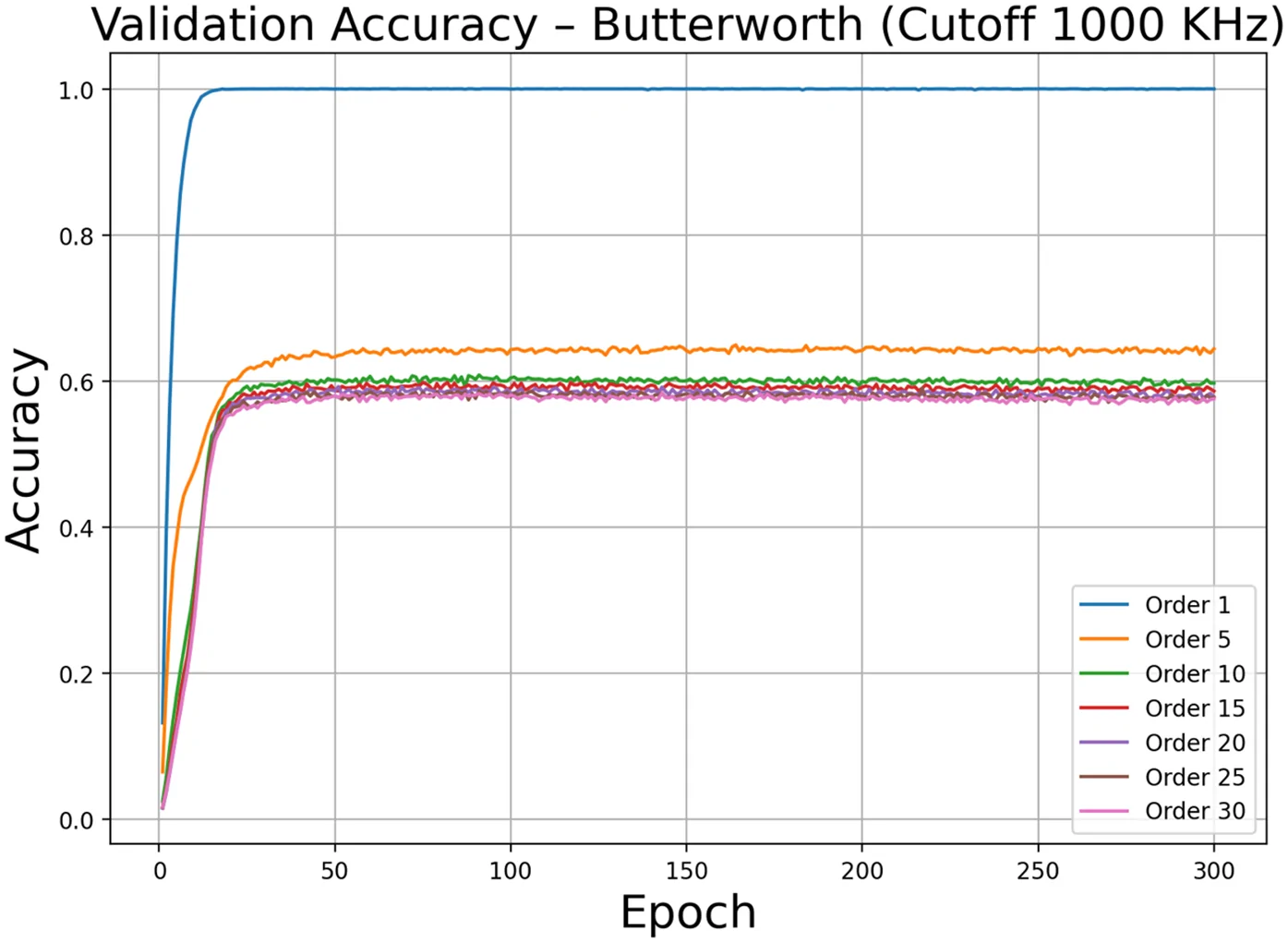
MLP validation accuracy vs. filter order (Butterworth, 1 MHz cutoff).

#### 4.6.2 Chebyshev low-pass filter.

To further test the effectiveness of filtering as a defense, we applied a first-order Chebyshev low-pass filter to 2,000 training traces and then varied the cutoff frequency and filter order. The results are shown in Table 11 and Fig 19. With a first-order filter, the outcome was much like that of the Butterworth filter: it removed only a small amount of noise, which improved the SNR and allowed the model to converge more quickly, but it did not meaningfully reduce the attack’s success. At a cutoff frequency of 1 MHz, raising the order to 5, 10, and 15 reduced the model’s accuracy to 59.7%, 57.3%, and 56.8%. By comparison, the Butterworth filter gave slightly higher accuracies of 64.9%, 60.8%, and 59.8% at the same orders. The difference of about 3% to 5% reflects a trade-off in the Chebyshev design: by allowing a small ripple of 1 dB in the passband, it achieves a steeper drop-off in the stopband. This steeper slope is more effective at cutting out high-frequency leakage signals that are closely tied to the random information being processed. Once the order reaches 20 or above, however, both filters show little additional improvement, while the cost and complexity of implementation increase sharply. A different perspective comes from fixing the filter order at five and gradually lowering the cutoff frequency from 1 MHz to 500 kHz. In this case, the model accuracy steadily decreased, from 59.7% to 58.1%, 52%, 42.2%, 28.4%, and finally 16.1%. These results show that the Chebyshev filter is more effective at reducing noise than the Butterworth filter when used at lower cutoff frequencies. This shows that the Chebyshev design can be a better tool for reducing side-channel leakage, especially when maintaining low-frequency fidelity is not important and resources are limited.

**Table 11:** Chebyshev Type-I LPF: Validation accuracy by cutoff frequency and filter order.

Cutoff Frequency	1	5	10	15	20	25	30
	%						
1 MHz	100	59.7	57.3	56.8	57.2	56.9	56.8
900 kHz	100	58.1	53.7	52.9	52.8	52.2	52.1
800 kHz	100	52	45.6	44.1	43.6	43	42.9
700 kHz	100	42.2	35.1	33.6	33.3	32.9	32.8
600 kHz	100	28.4	23.6	22.3	22.6	21.6	22
500 kHz	100	16.1	10.5	10.5	9.8	9.5	9.3

**Fig 19:**
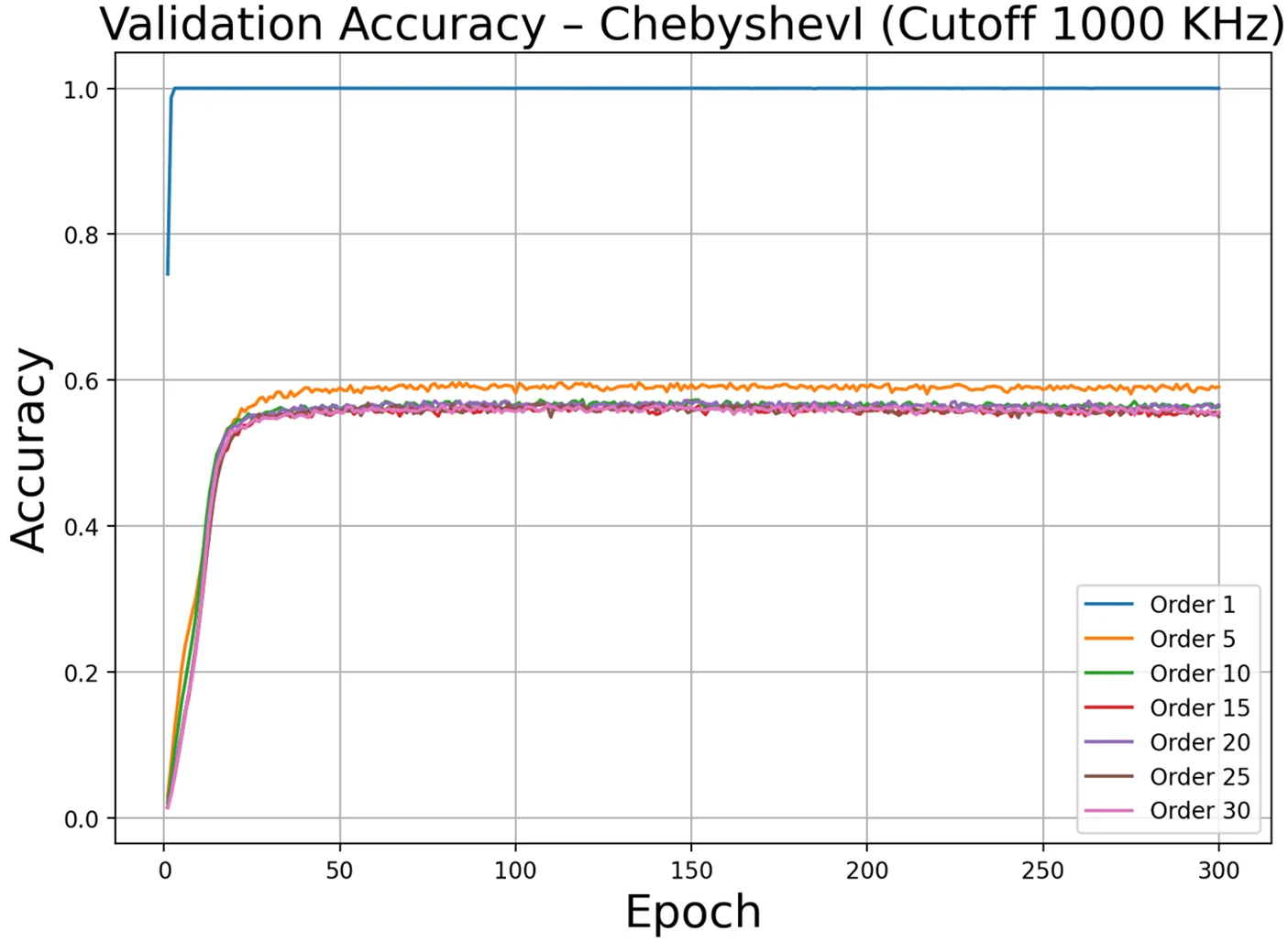
MLP validation accuracy vs. filter order (Chebyshev Type-I, 1 MHz cutoff).

### 4.7 Frequency-domain leakage characterization

To identify a frequency range with more distinguishable signal characteristics from the measured ML-KEM power traces, this study first applies the Fast Fourier Transform (FFT) for frequency-domain analysis. FFT is an efficient algorithm for computing the Discrete Fourier Transform (DFT), which transforms a time-domain signal into its frequency-domain representation. Through this transformation, the energy distribution of the measured power traces across different frequency components can be observed. Compared with directly analyzing the entire trace in the time domain, FFT provides a useful preprocessing approach for identifying candidate frequency bands where signal energy is more concentrated or where prominent spectral components appear.

The use of frequency-domain analysis for side-channel feature exploration is supported by previous studies. Mateos and Gebotys proposed Correlation Frequency Analysis (CFA), showing that side-channel information is not uniformly distributed across all frequencies and that some frequency components are more likely to leak information related to the processed data [32]. Their work demonstrates that power or EM traces can be transformed into the frequency domain by FFT, and that selected frequency components can be used for side-channel analysis. Chawla et al. further combined multi-band narrow band-pass filtering with CFA, showing that isolating specific frequency bands can improve the observability of side-channel signals and enhance the effectiveness of frequency-domain analysis [33]. In addition, Meynard et al. characterized EM side-channel leakage in the frequency domain and showed that sensitive information can be concentrated in specific frequencies or bandwidths, rather than only appearing at the clock frequency or its harmonics [34]. These studies provide methodological support for using FFT as a preliminary tool to search for candidate frequency bands in side-channel analysis.

Based on this motivation, this study applies FFT to 2,000 ML-KEM power traces collected from our measurement setup and computes the average FFT spectrum. Fig 20 shows the average spectrum within the frequency range from 0.5 MHz to 1 MHz. As shown in the figure, this frequency range does not exhibit a flat spectral distribution. Instead, several prominent spectral peaks and energy-concentrated regions can be observed. For example, noticeable peaks appear around approximately 0.54 MHz, 0.65 MHz, 0.76 MHz, 0.86 MHz, and near 0.98 MHz. Some of these peaks reach approximately 35 dB to 40 dB. Moreover, the average spectral energy from around 0.75 MHz to 0.95 MHz is relatively higher than that of the lower part of the observed range, suggesting that this band contains stronger periodic power components or hardware switching-related signal characteristics.

**Fig 20:**
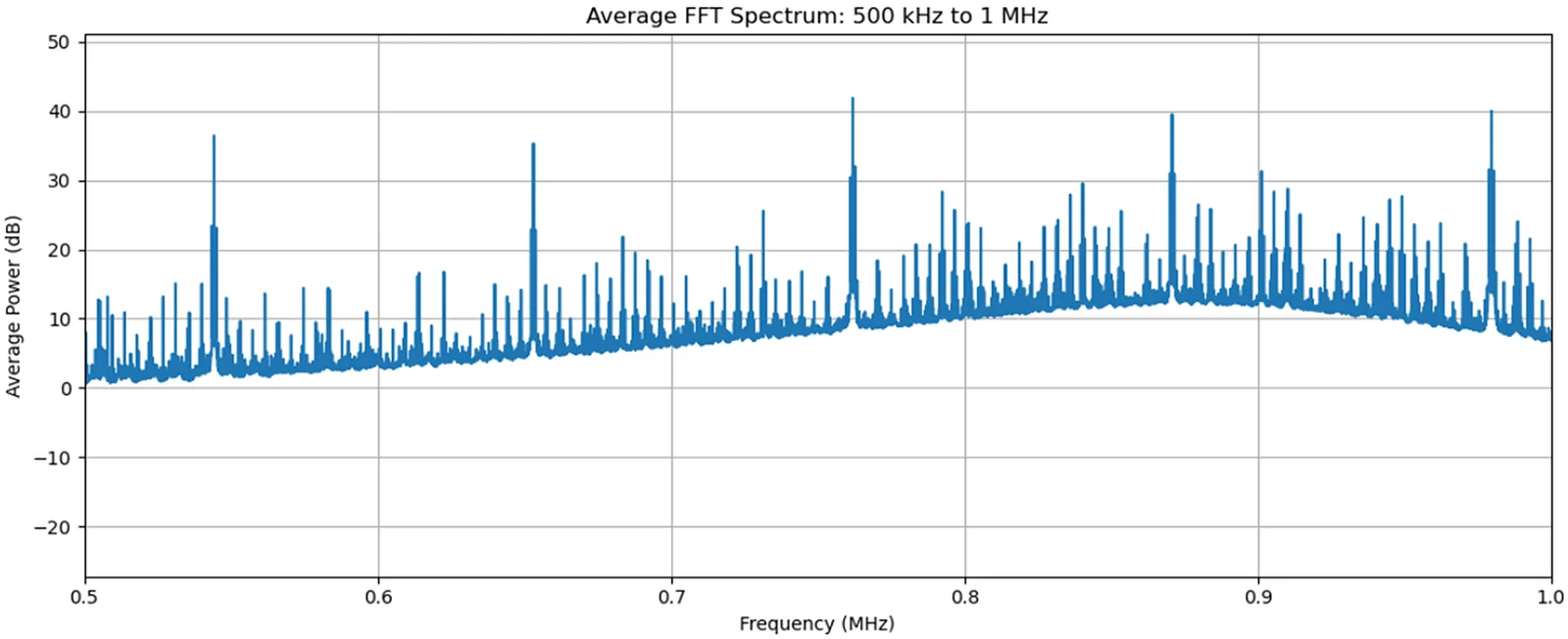
Average FFT spectrum of 2,000 ML-KEM power traces in the frequency range from 0.5 MHz to 1 MHz.

Therefore, this study selects the frequency range from 0.5 MHz to 1 MHz as the target band for subsequent analysis. This selection is motivated by previous frequency-domain side-channel studies, which show that specific frequency components or bands may carry more distinguishable side-channel information, and is further supported by our FFT results obtained from 2,000 measured ML-KEM power traces. As shown in Fig 20, the 0.5 MHz to 1 MHz range contains several prominent spectral peaks and energy-concentrated regions. Based on these observations, this band is adopted for the following filtering and side-channel attack experiments.

## 5. Conclusion

This study systematically investigated the side-channel vulnerability of the NIST-standardized post-quantum cryptographic scheme ML-KEM implemented on an ARM Cortex-M4 microcontroller, a representative platform for resource-constrained embedded systems. Although ML-KEM provides strong algorithmic security against quantum adversaries, our results show that its practical implementation can still be highly vulnerable to deep-learning-based side-channel attacks. In particular, an MLP-based profiling model achieved 99.98% byte-level accuracy in recovering the 32-byte encapsulated message from a single power trace, demonstrating that single-trace message recovery against embedded ML-KEM is feasible in practice. In addition, cross-device experiments on three extra STM32F415 microcontrollers showed that the learned leakage features remain highly transferable at the byte level, while full-message recovery is more sensitive to device-level variation. These findings indicate that implementation-level leakage in embedded ML-KEM is not only strong, but also sufficiently stable to pose a realistic threat beyond a single laboratory device.

To further understand the nature of the exploitable leakage, this work also conducted a frequency-band sensitivity analysis using Butterworth and Chebyshev low-pass filters under different cutoff frequencies and filter orders. The results show that attenuating specific spectral components can substantially reduce attack accuracy, suggesting that a significant portion of the learnable leakage resides in higher-frequency bands. Rather than interpreting offline digital filtering as a standalone practical defense, these observations should be viewed as evidence of the spectral dependence of DL-based side-channel attacks, which can help guide the design of realistic countermeasures in future work. Overall, this study highlights that evaluating post-quantum cryptographic implementations requires not only algorithmic security analysis, but also careful consideration of device-level leakage behavior, attack transferability, and the frequency characteristics of physical side-channel information.
